# Contagion and escalation of rule violations

**DOI:** 10.1098/rspb.2026.1940

**Published:** 2026-10-21

**Authors:** Lucas Molleman, Karlijn Hoyer, Gerben A. van Kleef, Christin Scholz, Jan Hausfeld

**Affiliations:** ^1^ Department of Psychology, University of Amsterdam, Amsterdam, The Netherlands; ^2^ Department of Communication Sciences, University of Amsterdam, Amsterdam, The Netherlands; ^3^ Department of Microeconomics, University of Amsterdam, Amsterdam, The Netherlands

**Keywords:** rule-following, social influence, group dynamics, rule violations, social order

## Abstract

Human societies rely on rules to coordinate behaviour, sustain cooperation and maintain social order. Yet compliance can be fragile when rules are weakly enforced and individuals observe others violating them. Prior research shows that rule violations and other antisocial behaviours can spread through social influence, but less is known about how the severity of violations shapes these dynamics. In many real-world settings, rule-breaking occurs in degrees, from minor infractions to major violations, and individuals may not only imitate others but sometimes exceed the violations they observe. Here, we combine simulations and a behavioural experiment (*n* = 750) to study how the magnitude of observed rule violations shapes subsequent violations, and how this can generate escalation dynamics. Our simulations show that when individuals occasionally escalate beyond observed violations, even rare escalation can trigger a rapid shift from widespread compliance to collective rule-breaking. In our pre-registered experiment, observing major violations strongly increased rule-breaking, especially when committed by socially close others. Observing minor violations prompted many participants to follow suit and a minority to escalate beyond the violations they observed. Parameterizing the simulations with these behavioural responses shows that such escalators can disproportionately undermine collective compliance. These results highlight how asymmetric responses to rule violations and rare escalatory behaviour can transform small deviations into large-scale breakdowns of rule compliance.

## 1. Introduction

Rule-following is a central feature of human societies and a key mechanism for coordinating behaviour in groups. Rules, including formal laws and regulations as well as informal social and moral norms, prescribe or proscribe particular behaviours, thereby creating the predictability needed for cooperation, social order and collective action [1–5]. Understanding when and why individuals follow or violate rules is therefore a fundamental question across the biological, behavioural and social sciences, including evolutionary biology, behavioural ecology, economics and psychology [6–17]. People often comply with rules voluntarily, even when doing so goes against their immediate self-interest and when enforcement is weak [6,11,12,15,16]. For example, people often queue in public spaces, avoid littering and report taxes honestly despite limited formal oversight. However, such voluntary rule compliance is fragile: observing others violate rules can give rise to behavioural contagion, whereby rule violations spread through social influence, potentially undermining cooperation, eroding social order and contributing to undesirable outcomes such as vandalism, rioting and group bullying [4,6,11,16,18–21].

In many real-world settings, rule violations are not simply binary but vary in severity: individuals may deviate slightly or substantially (e.g. driving at 75 vs. 100 mph in a 70-mph zone). While existing theoretical and experimental approaches have treated rule-following as a graded behaviour, less is known about how the severity of observed violations shapes subsequent behaviour, and whether such responses can trigger escalation, where individuals exceed the violations they observe.

In this paper, we examine how rule violations spread through social influence and, in particular, how escalation of rule violations can emerge when individuals respond to others’ violations. We combine simulations with a behavioural experiment to study situations in which rule adherence varies in degree, from full compliance to minor infractions and more severe violations. Our simulations show how responses to such graded behaviour can scale up to collective dynamics: when individuals occasionally exceed the violations they observe, even rare escalatory responses can trigger rapid transitions from widespread compliance to collective rule-breaking. We test these dynamics in a preregistered experiment in which participants repeatedly decide how strictly to follow a costly rule after observing the behaviour of another individual. We show that observing violations increases rule-breaking—especially when committed by socially close others—and that a minority of participants escalate beyond the violations they observe. Simulations parameterized with these behavioural responses reveal that such escalators can disproportionately undermine collective compliance.

Research on rule-following and social norms has generated important insights into when individuals comply with or violate rules and how social influence shapes behaviour [19,22–24]. Experimental paradigms typically frame behaviour in terms of rule compliance versus violation, for example, by asking participants to wait at a traffic light or crossing, choose a rule-consistent versus payoff-maximizing option or turn left or right at a road fork [11,25,26]. These studies show that observing others’ behaviour can shift individuals’ rule-following in the direction of the observed behaviour, with observed rule violations often exerting a stronger influence than observed rule-following [11,25,27], consistent with findings from related domains such as honesty and prosociality [28–31]. More generally, both theoretical and empirical work has shown how social influence can lead individuals to converge towards the behaviour of others, for instance, in collective actions such as cooperation, protest participation or norm adherence [19,22].

At the same time, many real-world rule violations occur in degrees rather than as all-or-nothing decisions [32–34]. For example, speeding in traffic ranges from adhering to limits to slight speeding to reckless racing; plagiarism ranges from paraphrasing without citation to outright copying and tax evasion ranges from minor underreporting to large-scale fraud. Some theoretical models represent behaviour as a gradual action space in which social influence shapes dynamics within a bounded range of possible behaviours [35–37], and some experimental paradigms likewise allow participants to vary the extent to which they follow a rule [38,39]. However, less is known about how the magnitude of observed violations shapes the magnitude of subsequent violations. In particular, existing work has focused on imitation and convergence, largely overlooking the possibility that individuals may respond by exceeding the violations they observe. Such responses could generate escalation dynamics, whereby small initial deviations progressively expand the range of acceptable behaviour, for example, minor speeding may encourage others to drive even faster, just as shouting and gesturing can escalate into violent action [20,40–42]. To address this, we model rule-following as a graded behaviour and examine how individuals respond to observing different levels of rule violation. This allows us to distinguish between the spread of violations through imitation and their escalation when individuals exceed the behaviour they observe.

Our objective is to understand how observed behaviour changes individuals’ own rule-following. We focus on situations in which individuals face an immediate incentive to deviate from a rule (such as saving time by speeding or jaywalking, gaining financially through tax evasion or avoiding effort by cutting corners) while enforcement is absent or weak, and compliance therefore depends on individuals’ willingness to follow the rule voluntarily. By abstracting away from punishment, moral considerations and other contextual factors, we isolate the influence of observing others' behaviour on rule-following, complementing the extensive literature on sanctions and enforcement [11,43,44]. Accordingly, our treatment comparisons are designed to identify how observing different examples of rule-following and rule violation shapes subsequent behaviour, irrespective of the psychological processes through which participants integrate the rule, incentives and social information. Although our paradigm does not reproduce the full complexity of real-world rule-governed behaviour, where rules often promote cooperation and protect collective welfare and violations may entail punishment or other consequences for oneself or others, it captures an important class of situations in which compliance depends primarily on voluntary rule-following.

Social influence is widely considered to be shaped by social proximity: people are generally more influenced by similar or ‘ingroup’ members than by dissimilar or ‘outgroup’ members, because ingroup behaviour provides more relevant information [45–47] or because individuals are more motivated to fit in and avoid disapproval [1,48–50]. Across domains, evidence shows stronger influence from socially close others [29,51–56], yet it remains less clear how social proximity shapes responses to observing socially desirable versus socially undesirable behaviour [26,29,30,57]. Recent studies on prosocial decision-making indicate that selfish choices are influential regardless of social proximity, whereas generous choices primarily influence behaviour when they come from socially close others [28]. This suggests that the asymmetry in influence between generous and selfish examples is smaller for socially close than for socially distant others. Whether a similar pattern extends to rule-following remains unknown. Based on these findings, we hypothesized that rule-following examples from socially close others would likewise more strongly promote rule compliance than rule-following examples from distant others, thereby reducing the asymmetry between rule-following and rule-violating examples. If this holds true, rule-following examples from socially close others may play a crucial role in curbing the spread of rule violations.

We proceed in three steps. First, we develop a simulation model to illustrate how social influence shapes collective rule-following when individuals observe others following a rule or committing minor or major violations. This model aims to build intuitions about the possible long-term consequences of different behavioural responses. Second, we present a pre-registered behavioural experiment in which participants decide how strictly to follow a rule after observing the behaviour of others, varying both the rule-related behaviour and social proximity of these others. Third, we use the experimentally measured individual responses to simulate how they might scale up to collective patterns of rule compliance or rule violation. Both the simulations and the experiment are guided by the hypotheses that (i) rule-violating examples exert stronger influence than rule-following ones, and that (ii) social proximity increases the influence of rule-following examples, thereby reducing the asymmetry between rule-following and rule-violating examples.

## 2. Results

### (a) Simulations

#### (i) Setup

To explore how social influence shapes the dynamics of rule-following and rule violation on a graded scale, we developed a simulation model in which agents update their level of rule-following over time. The aim is to map a possibility space of group-level consequences that emerge from different patterns of individual responses, rather than predict precise real-world outcomes. Specifically, we focus on how social influence can transform an initially orderly population—in which most agents follow rules—into one where rule violations may arise and spread. We examine how different (symmetric or asymmetric) reactions to observing rule-following and -breaking scale up to collective dynamics. To this end, we keep the model simple and abstract away from cognitive mechanisms and payoff consequences of behaviour. Here, we describe the general simulation procedures; for implementation details, see §4 and the Supplementary Methods.

The simulated population consists of 100 agents, each choosing a value between 0 (strict rule-following) and 1 (complete rule-breaking), with intermediate values representing limited rule-breaking. At initialization, agents’ behaviours are initially drawn from a normal distribution with mean 0 and s.d. 0.5, and are subsequently winsorized at 0 and 1. This reflects a population where most agents follow rules, but some commit minor rule violations.

At each time step, a randomly selected ‘focal’ agent observes another randomly selected ‘example’ agent, and updates their behaviour according to *r_t_*_+1_ = (1 − *s*) * *r_t_* + *s* * *X*, where *s* is the strength of social influence, and *X* is the example's behaviour. Across simulations, we vary *s* depending on whether the example is more rule-following (*X* < *r*) or more rule-violating (*X* > *r*) than the focal agent, and whether the example is socially close or distant.

To sketch the space of possible collective dynamics and outcomes, we simulate scenarios that differ in how agents respond to the example's rule-related behaviour. These scenarios build on each other: starting with established settings in which (i) rule-violating examples exert stronger influence than rule-following examples [11,28–30], and (ii) social proximity strengthens the influence of both types of examples [51–56]. We then consider more nuanced scenarios in which (iii) the asymmetry between rule-following and rule-violating examples is present only when others are socially distant [28,29], before ending with a hitherto unexplored scenario in which (iv) agents occasionally escalate by exceeding the rule violations they observe.

#### (ii) Group dynamics

In a baseline scenario where social influence is symmetric (i.e. *s* is constant regardless of the example being rule-following or rule-violating), the population's average rule-following remains stable (figure 1A, red diamonds). However, when updating is asymmetric, such that *s* is higher in response to rule-violating examples than to rule-following ones, rule violation increases over time and stabilizes at intermediate levels (figure 1A, green dots). This increase in rule violation levels off because agents' updates are bounded: the social influence parameter *s* is never greater than 1, and agents cannot become more rule-breaking than the most extreme behaviour present at the start of the simulation.

We next examine the role of social proximity. We build on the baseline scenario, retaining the assumption that rule-violating examples exert stronger influence than rule-following examples. In one condition, agents are socially close to each other, reflecting homogeneous groups where others belong to an ingroup. In the other condition, others are socially distant, reflecting interactions in heterogeneous groups where others belong to an outgroup. If social proximity increases the strength of both rule-violating and rule-following examples, we observe that rule-breaking is higher in socially close groups (figure 1B; purple triangles). When we assume that rule-violating examples only exert stronger influence than rule-following ones when others are socially distant, this pattern flips (figure 1C).

Finally, we explore a scenario where rule-breaking can escalate (figure 1D). With probability *e*, agents escalate others’ rule violations, adjusting with a strength *s_e_* > 1, reflecting exceeding observed rule violations. With the complementary probability (1 − *e*), agents adjust with strength *s_¬e_* = (*s* − *s_e_* * *e*)/(1 − *e*), keeping average social influence constant across simulations. In this case, initially minor rule violations are rapidly amplified, and groups converge on complete rule-breaking (figure 1D, purple dots and line).

Together, these simulations show how collective rule-breaking can escalate through social influence, even if it starts with minor violations. The outbreak of rule violations is especially likely when agents respond more strongly to rule-violating than rule-following examples, and when socially close others exert stronger influence. In the next section, we present an experiment to test these mechanisms, examining how people respond to others’ rule-following, and varying degrees of rule violation, and whether these responses depend on whether the observed others belong to a real-world ingroup or outgroup.

### (b) Behavioural experiment

#### (i) Setup

To test how people respond to others' rule-following, and (minor and major) rule violations, we conducted a behavioural experiment. In the week before the 2024 US presidential election, we recruited 750 participants from the USA, roughly half of whom self-identified as Republicans and the other half as Democrats. They completed a task in which they were asked to follow a rule at a personal cost, and they could do so to varying degrees. Participants saw an animated ‘shooting star’ moving across a row of 100 squares while first gaining and then fading in intensity. Participants were told that the rule was to ‘select the square that lit up most intensely’ (figure 2A). This most-intensely lit cue square was always located somewhere near the middle of the row. However, choosing a square farther to the right yielded higher monetary rewards, creating a trade-off between rule-following and self-interest: moving rightwards yielded higher rewards but greater deviation from the rule, whereas moving leftwards reduced payoffs and thus did not constitute a self-serving violation. This design allows us to capture graded, payoff-motivated rule violations analogous to exceeding a speed limit or underreporting income.

After making a first choice, participants observed the choice of a prior participant who saw the same stimulus, providing descriptive information about others’ compliance with or violation of the rule. We preselected this choice from a pretest experiment to reflect either rule-following, minor violations or major violations (see §4 and the Supplementary Methods). The prior participant's choice was displayed on the same row of squares as a marked square indicating where they had clicked (figure 2A, bottom; ‘other's choice’). This marker remained visible on the screen while participants made their second choice, allowing them to directly compare their own decision with the observed behaviour. Rule-following examples involved a choice near the cue; major violations involved examples selecting the rightmost square; and minor violations fell roughly one-third of the way between the two (e.g. around square 66 if the cue was at square 50). After observing this choice, participants made a second selection. Mirroring the simulations, responses were normalized such that 0 represented full rule compliance (the cue), and 1 represented full rule-breaking (the rightmost square). On this normalized scale, the examples committing minor violations were located around 0.33.

To examine the role of social proximity, the observed other was either a self-identified Democrat or Republican. A manipulation check confirmed that participants felt much closer to members of their own party than the opposing party (Supplementary Methods; figure S2). Each participant completed six trials in randomized order, varying in the observed behaviour (rule-following, or committing minor or major rule violations) and social proximity (close, i.e. same party, or distant, i.e. other party), in a within-subject design. This design mirrors the simulation framework by allowing us to estimate how individuals respond to different levels of rule-related behaviour under varying social proximity. In particular, it can capture asymmetric responses to rule-following and rule-violating examples, and the possibility that initially compliant individuals exceed observed violations, providing key inputs for the dynamics explored in our simulations. Unlike the simulations, the experiment measures individual responses to a single observed example rather than emergent group dynamics. The simulations then use these measured responses to explore how such individual updating could scale up to collective outcomes.

#### (ii) Analysis

By comparing choices before and after observing those of others, we first assess the commonly studied cases of seeing others follow rules or commit major violations—with examples set at normalized levels of 0 and 1—and how the social proximity of the other moderates behaviour. We then examine responses to minor violations. We further quantify the strength of social influence (*s*) as the relative adjustment towards the other's choice (matching the simulations; §4). We do so for each treatment, and for cases where the other was either more rule-following (*X* < *r*_1_) or more rule-violating than the participant's own first choice (*X > r*_1_). Unless otherwise stated, all analyses use linear mixed-effects models with random intercepts for participants. This approach accounts for repeated measures and yields reliable estimates that are generally robust to distributional assumptions [58]. The resulting regression coefficients can be directly interpreted as effect sizes (i.e. absolute mean differences in normalized responses). All tests are two-sided. For pre-registration, see [59].

#### (iii) Initial choices

Before observing others’ behaviour, participants already showed substantial degrees of rule violation. On average, these first choices deviated markedly from the cue value (mean normalized choice = 0.341; *t* = 24.86, *p* < 0.001), with wide variation (figure 2B, grey ridge on top). Outright rule-breaking—choosing the square with the highest payoff—occurred in 22.7% of all these choices. The same qualitative patterns were already evident in trial 1 of the task (before any spillovers from other treatments could have occurred; mean = 0.323; 19.1% full rule-breaking; supplementary material, figure S3), and varied little by gender, age or political affiliation (supplementary material, table S1).

#### (iv) Rule-violating examples exert stronger influence than rule-following ones

Observing others who followed the rule led to only a slight reduction in rule-breaking, if any (figure 2B, blue dots). Average rule violation after rule-following examples was 0.004 higher than the overall baseline first choices calculated across all trials. Within-trial changes were small but negative (mean change = −0.013; *t* = −2.075, *p* = 0.038). By contrast, observing major rule violations had a strong effect: participants substantially increased their degree of rule-breaking (figure 2B, red dots; change from overall baseline = +0.174; mean within-trial change = +0.148; *t* = 16.160, *p* < 0.001). This asymmetry cannot be explained by a floor effect: although many participants initially chose to follow the rule, about one quarter of participants initially chose full rule-breaking, so there was ample variation at both ends.

The preregistered linear mixed-effects model further confirmed this asymmetry. Second choices were more strongly influenced by rule-violating than rule-following examples (table 1, Model 1; *z* = 21.95, *p* < 0.001), supporting the hypothesis that social influence is asymmetric: rule-violating examples exert more influence than rule-following ones. Consistent with the by-treatment analysis, when the example was more rule-following than the participant's first choice (i.e. *X* < *r*_1_), average updates were minimal (mean = −0.038). But when social information was more rule-violating (*X* > *r*_1_), updates were large (mean = +0.160; Wald test comparing absolute effect sizes: *z* = 25.47, *p* < 0.001).

**Table 1.** Hypothesis tests. Shown are estimates (with standard errors in brackets) from linear mixed models fitted to second choices (after observing social information), with ‘participant’ as randomly varying intercept. Note that each model also includes the participants’ first choices in each trial, but contrary to the registered model, we fixed the slope of this predictor to 1, to allow for comparisons between the coefficients for rule-violating and rule-following examples. In the bottom three rows, social proximity is modelled (abbreviated as ‘sp’) as a binary variable (the example of an ‘other’ from the other party is coded as 0, and from the same party coded as 1). These results are confirmed using continuous measures of perceived social proximity from the inclusion of the other in the self-scale (supplementary material, table S2). For the preregistration, see [59].

predictor	model 1	model 2	model 3
following example (baseline)	*b* = −0.012 (0.008), *t* = −1.559, *p* = 0.119	*b* = −0.002 (0.010), *t* = −0.159, *p* = 0.873	*b* = −0.002 (0.009), *t* = −0.179, *p* = 0.858
major violation example	*b* = 0.186 (0.009), *t* = 19.914, *p* < 0.001	*b* = 0.149 (0.013), *t* = 11.333, *p* < 0.001	*b* = 0.150 (0.012), *t* = 12.925, *p* < 0.001
minor violation example	–	–	*b* = 0.056 (0.012), *t* = 4.858, *p* < 0.001
social proximity (sp)	–	*b* = −0.021 (0.013), *t* = −1.629, *p* = 0.103	*b* = −0.021 (0.012), *t* = −1.851, *p* = 0.064
major violation example × sp	–	*b* = 0.074 (0.0187), *t* = 3.968, *p* < 0.001	*b* = 0.074 (0.016), *t* = 4.494, *p* < 0.001
minor violation example × sp	–	–	*b* = 0.016 (0.016), *t* = 0.986, *p* = 0.324

#### (v) Social proximity amplifies the effect of rule-violating examples

Contrary to our expectation, higher social proximity did not reduce the asymmetry between rule-following and rule-violating examples. Instead, it increased it. The interaction in table 1 (model 2) indicates that the difference between responses to rule-following and rule-violating examples depended on social proximity. Inspection of figure 2B shows that this interaction was driven primarily by stronger responses to rule-violating examples from socially close others, whereas, contrary to our prediction, responses to rule-following examples remained small regardless of social proximity. Participants showed more rule-breaking after observing someone fully breaking the rule from their own political group than from the opposing group (table 1, model 2). This suggests that social proximity amplifies, rather than buffers, the stronger influence of rule-violating examples.

#### (vi) Impact of observing minor rule violations

Seeing others commit minor rule violations led to a moderate increase in rule-breaking behaviour (figure 2B, orange dots; raw change: +0.061; mean within-trial change: +0.052; *t* = 9.467, *p* < 0.001). In this condition, participants observed others’ choices roughly one-third of the way between full compliance and outright rule-breaking. On average, participants did not exceed the level of (minor) rule violations they observed. The average shift (+0.05) was proportionate—about one-third the effect size of observing major rule violations—indicating, at this aggregate level, no evidence for a nonlinear escalation or ‘slippery slope’ (cf. figure 1D). All observed effects were already present in trial 1 of the task, in which participants have only seen a single other choice (supplementary material, figure S3).

#### (vii) Individual updating of behaviour

Although average behaviour remained bounded after observing minor rule violations, a closer look at individual updates reveals that in 4.4% of trials participants escalated beyond the observed degree of rule-breaking, exceeding the violation they observed (involving 33 participants overall; figure 3A, purple filled triangles). In 58% of trials, participants did not update their choices after observing another participant. About 25% of trials involved partial adjustment to the social information, such that the second choice was a compromise between the first choice and the social information (0 < *s* < 1). Exact matching (*s* = 1) occurred in 11%–22% of trials, most often when observing a major rule violation (22%). Consistent with earlier results, these social adjustments were slightly larger when participants observed someone from their own political group (supplementary material, figure S4), suggesting that social proximity modestly amplifies responsiveness across all types of examples.

**Figure 1.**
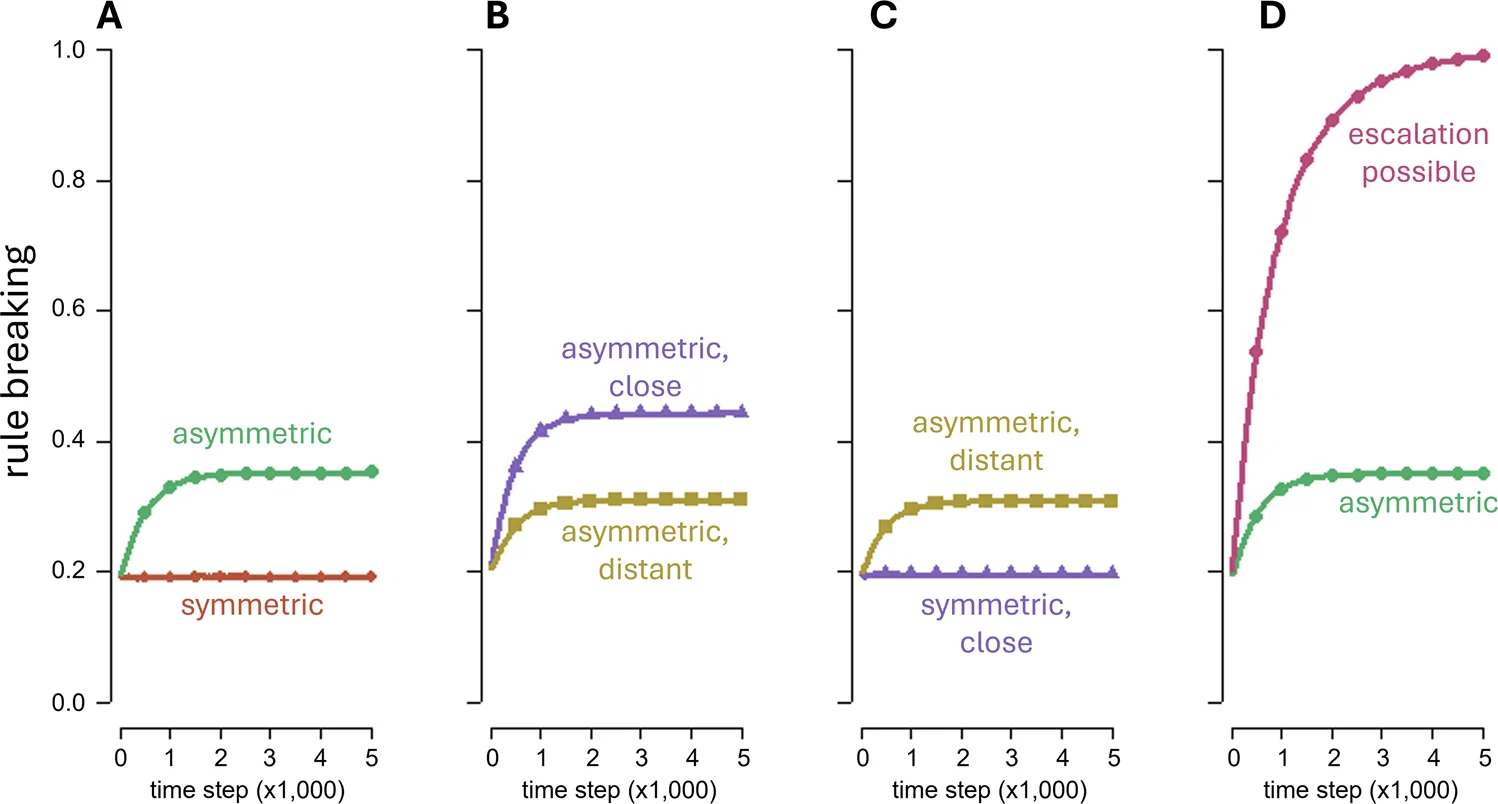
Simulated group dynamics of rule-breaking. Lines show mean rule-breaking across 30 simulation runs. (A) Symmetric influence of rule-following and rule-violating examples maintains initial levels of rule-breaking (red diamonds), whereas stronger influence of rule-violating examples increases rule-breaking (green diamonds). (B) When social proximity strengthens social influence, this asymmetric effect is exacerbated in socially close groups (purple triangles), and attenuated in socially distant groups (golden squares). (C) When social proximity strengthens influence of rule-following examples—such that rule-following and rule-violating examples are more symmetric—rule-breaking is highest in socially distant groups. (D) When agents can ‘escalate’ observed rule violations—exceeding others’ transgressions, rule-breaking rapidly intensifies and results in collective rule-breaking (see §4 and the Supplementary Methods for implementation details). The asymmetric condition from panel (A) is shown for comparison (green dots). See figure S1 in the supplementary material for a version including individual simulation runs.

**Figure 2.**
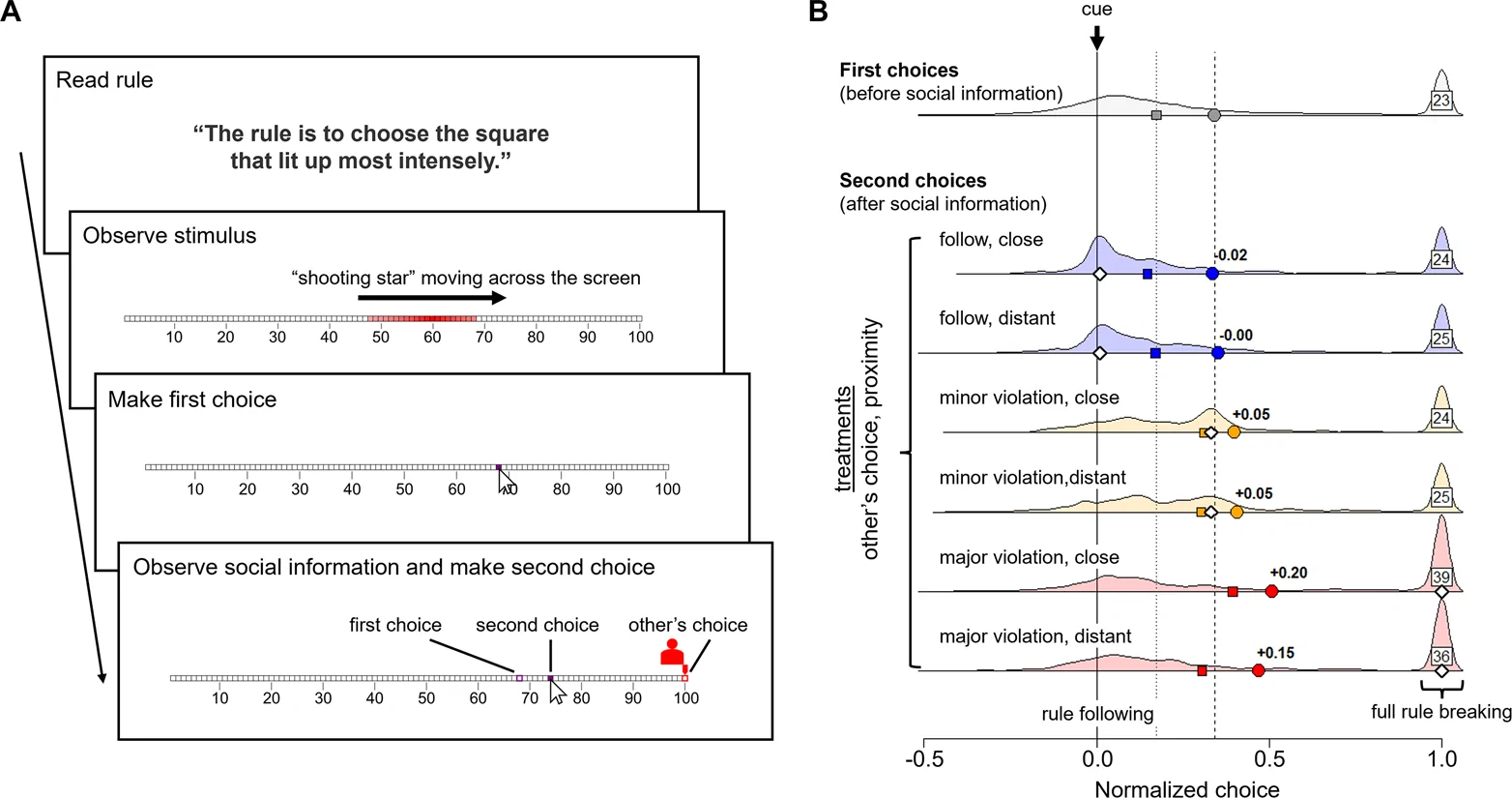
Experimental task and main results. (A) Participants observed an animated ‘shooting star’ moving across 100 squares. The rule was to select the square that lit up most intensely (the ‘cue’ square), whereas choosing a square farther to the right increased earnings (§4). Participants made a first choice (purple fill at the cursor). Then, they observed the choice of a previous (‘other’) participant who was labelled as either a Democrat or a Republican (red bordered square below the avatar). This social information, together with the participant's own first choice (purple bordered square), remained visible on the screen while participants made their second choice. (B) Distributions of first and second choices. Second choices are shown by treatment, varying the other's behaviour (rule-following, minor violation, major violation) and social proximity (as reflected by their political identity). White diamonds indicate mean social information, filled coloured circles indicate participants’ mean second choices (labels show mean changes from first choices) and filled coloured squares indicate medians. First-choice means and medians are shown in grey, with thin vertical reference lines for comparison. Numbers above *x* = 1 indicate percentages of full rule breakers.

**Figure 3.**
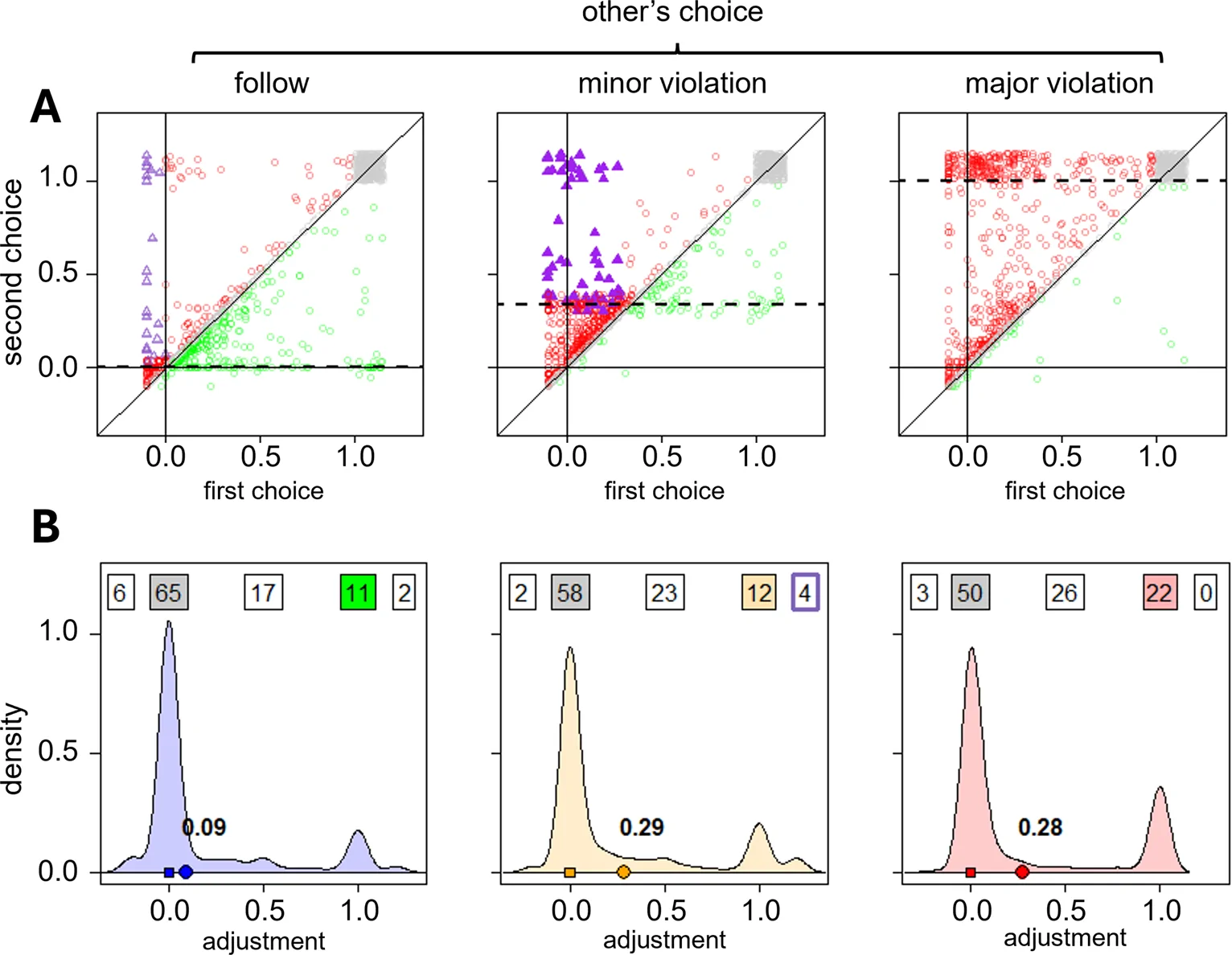
Updating from first to second choices for three types of social examples. (A) Scatterplots show second choices as a function of first choices when the observed other participant either followed the rule, or committed minor or major violations. Dots show participants’ choices in individual trials. Colours indicate the types of updates (green: reduced rule-breaking; red: increased rule-breaking; grey: no change; purple: updates exceeding the observed behaviour (*r*_1_ < *X* and *r*_2_ > *X*). In the rule-following condition, open triangles denote participants whose first choice lay left of the cue and whose second choice overshot the observed rule-following example; in the minor violation condition, filled triangles denote ‘escalation’ beyond the observed rule violation. Thick horizontal dashed lines show average social information (*X*) in each treatment. Thin lines at *x* = 0 and *y* = 0 mark the cue; the diagonal indicates no change. First or second choices that are full rule-breaking (*x* = 1 and *y* = 1) are shown with positive-only jitter to reduce overplotting. (B) Distributions of adjustments from first to second choices calculated as *s* = (*r*_2_ − *r*_1_)/(*X* − *r*_1_). Circles with labels indicate average adjustments, and squares indicate medians. For visual presentation, we winsorized the data at −1.2 and 1.2. Percentages of qualitatively different adjustment types are shown up top, with from left to right: moving away from social information (*s* < 0), staying (*s* = 0), compromising (0 < *s* <1), copying (*s* = 1) and escalating (*s* > 1). For a breakdown by treatment including low versus high social proximity, see figure S4 in the supplementary material.

We observe considerable individual differences in responses to social information. Roughly, 31% of participants never adjusted their initial choice across all six trials (average *s* = 0; supplementary material, figure S5). Furthermore, about 22%–24% consistently followed the rule and 18%–23% consistently broke it, depending on how consistent behaviour is defined (see supplementary material). Participants who initially chose mid-range values (0.1 < *r*_1_ < 0.9) showed the largest updates (supplementary material, figure S5).

#### (viii) Parameterized simulations

Finally, we use the experimentally estimated updating parameters to simulate group-level dynamics. These parameters exhibit asymmetric influence: participants were more responsive to examples that were more rule-violating (*X*  >  *r*_1_) than to examples that were more rule-following (*X*  <  *r*_1_). In the simulations, agents update their behaviour based on social information weighted by the empirically observed mean sensitivity (*s*). We assume that the one-off updates from the experiment extend to repeated updates, allowing us to examine the possible long-term consequences of the observed social influence effects. We use the same initial conditions as in the simulations from figure 1, such that most agents follow the rule, with some committing minor rule violations (see supplementary material, figure S7, for simulations with initial conditions based on the first choices from the experiment, confirming the main results reported below).

We first simulate a scenario in which agents update their choices based on the observed average responses to more rule-following and more rule-violating examples. Under these conditions, rule-breaking increases from low to moderate levels (figure 4, green diamonds), locating the group dynamics near the possibility space outlined in figure 1A (green line), where social influence amplifies deviation from the rule.

Introducing a small probability of escalation (*e*  =  0.044), as estimated from the experiment, further shifts the group into the escalation region (figure 1D, purple line labelled ‘escalation possible’): Although rare and seemingly innocuous at the individual level, such escalation, where agents occasionally go beyond the (minor) rule violations they observe, can tip the population into collective rule-breaking (figure 4, purple dots).

This effect is tempered when adding unconditional agents (who follow or break the rule regardless of what others do) in the proportions observed in the experiment. The group then stabilizes at an intermediate level of rule-breaking (figure 4, blue triangles), corresponding to a return towards the constrained divergence region of figure 1. Together, these simulations suggest that over time, social influence can drive the progressive spread of rule-breaking—especially when (small) escalations occur, but that the presence of unconditional rule-followers can stabilize behaviour at an intermediate degree of rule-breaking.

## 3. Discussion

Our study highlights the powerful role of social influence in shaping rule-following behaviour. In our experiment, we observed that rule-violating examples exerted a stronger influence than rule-following examples, and this effect was amplified by social proximity. Our data suggest that minor violations invite imitation and can sometimes trigger escalation. We then use the experimentally collected parameters to inform the simulations. We find that even when limited to a minority of individuals, escalatory responses can substantially undermine collective rule compliance as violations spread through groups. Together, our simulation model and behavioural experiment reveal how minor violations can lead to collective rule-breaking.

Our first hypothesis was that rule-violating examples have a stronger impact than rule-following examples. The data support this asymmetry (figure 2): observing rule violations strongly increased rule-breaking, whereas observing rule-following had little effect on compliance. This pattern mirrors recent findings in binary rule-following tasks [11], and aligns with prior research in prosociality, honesty and related domains [3,11–13], where examples showing socially undesirable behaviour were found to reduce socially desirable behaviour. Overall, our results suggest that social influence more readily exacerbates rule-breaking than it promotes rule-following. Importantly, this pattern was already evident in participants' first decision after observing another's behaviour (supplementary material, figure S3), indicating that the main effects do not depend on accumulated experience across repeated trials. The asymmetric social influence effects may help explain how collective rule-breaking—such as hooliganism, vandalism and tax avoidance—can gain traction and spread.

At the same time, our results reveal substantial heterogeneity in responses to social information. A notable fraction of participants consistently broke the rule, in line with payoff-maximizing behaviour given that violations increased earnings. More strikingly, a similarly sized group consistently followed the rule even after observing others break it. Such unconditional rule-following confirms recent findings [11,60] and may reflect that some individuals treat rules as deontic constraints (obligations to be followed regardless of payoffs or others’ behaviour [61]). By maintaining a baseline level of compliance, these individuals may help stabilize rule-following in social systems.

Our simulations show that even a small minority engaging in escalation, where minor violations lead to larger ones, can progressively increase rule-breaking. This suggests that in settings where escalation has harmful consequences, addressing minor violations early could help prevent their spread and amplification. However, this should not be taken to imply that all minor violations should be suppressed. Pushing boundaries can support learning and norm internalization in developmental contexts [62], while deviations from rules can foster experimentation and creativity [63,64]. The consequences of minor violations are therefore context-dependent.

Our design deliberately isolated how observing others' behaviour shapes voluntary rule compliance, while holding constant other determinants of rule-following such as punishment, externalities, repeated interaction and strategic interdependence. Accordingly, the rule was arbitrary and unenforced, payoffs were independent between participants, participants acted anonymously and decisions consisted of one-off responses to observed behaviour. These design choices also ensured close correspondence between the behavioural experiment and the simulation model, while abstracting away from important contextual factors that shape compliance in many real-world settings. Future studies could examine how such factors modify the observed social influence effects. For example, payoff interdependence may promote escalation [65], whereas punishment through disapproval or exclusion may suppress it; repeated interaction and the visibility of violations may further alter responses. Importantly, many everyday rules are weakly enforced, so voluntary compliance is an important feature of real-world rule-following rather than merely an experimental simplification. Nevertheless, our experiment is intended to identify how social influence changes rule-following, not to estimate the prevalence of rule violations in society. Absolute levels of rule-breaking depend on contextual factors such as enforcement and consequences, and may therefore be higher than in some real-world settings (e.g. speeding where enforcement is likely) but lower than in others (e.g. jaywalking at night).

Likewise, our simulations deliberately abstract away from the cognitive mechanisms that give rise to these behavioural responses. Individuals may respond to others' behaviour for many reasons, including informational conformity, uncertainty reduction, strategic adaptation, concerns about social approval or intrinsic respect for rules. Relatedly, we use the term ‘behavioural contagion’ descriptively to refer to the spread of behaviour through social influence, without implying that such transmission is passive or automatic [66]. Rather than modelling the underlying cognitive processes explicitly, we estimated behavioural updating directly from the experiment and used these empirically observed responses to examine their collective consequences. Future work could combine our framework with richer psychological models to investigate how specific cognitive mechanisms generate the behavioural responses documented here. Future research could also examine why minor violations arise and escalate, for example, whether small deviations allow individuals to gain while maintaining plausible deniability [67–69], or whether observing unpunished minor violations gradually changes perceptions of acceptable behaviour [25].

Our second hypothesis was that social proximity would mitigate the asymmetric effects of rule-following and rule-violating examples. We found the opposite: social proximity amplified this asymmetry. These results underscore the risks of echo chambers, where like-minded individuals may reinforce deviant behaviour. Our findings contrast with prior research on prosocial behaviour, where social closeness reduced the asymmetry between rule-following and rule-violating examples [28]. A likely reason is the abstract nature of our task: estimating a cue's position on a line, with a rule that is neutral and non-ideological. Because this rule was not tied to partisan identity, violations probably appeared as general breaches rather than as ingroup versus outgroup conflicts. As a result, even salient social identities had limited effects. Future work should explore whether social proximity plays a larger role when rules are tied to group identities or ideological divides.

Taken together, our study sheds light on the behavioural processes through which rule violations spread through social influence, showing how individuals' escalatory responses to minor rule violations can scale up to collective rule-breaking. These findings identify a behavioural mechanism through which minor rule violations may not only spread through groups, but also progressively amplify, increasing the severity of subsequent violations and ultimately giving rise to collective rule-breaking.

## 4. Methods

### (a) Simulations

We simulated a population of 100 agents making repeated choices on a scale from 0 (full rule compliance) to 1 (full rule-breaking). Initial choices were drawn from a normal distribution centred on 0 and winsorized at 0 and 1, producing a population in which most agents initially followed the rule while some committed minor violations. At each time step, a randomly selected focal agent observed another agent's behaviour and updated its choice according to *r_t_*_+1_ = (1 − *s*) * *r_t_* + *s* * *X*, where *s* reflects the strength of social influence. We allowed *s* to vary depending on whether the observed example was more rule-following or more rule-violating than the focal agent, and across levels of social proximity.

We used this framework to simulate the scenarios shown in figure 1, including symmetric versus asymmetric social influence of rule-following and rule-violating examples, variation in influence by social proximity and escalation in which agents could exceed observed rule violations. For figure 4, model parameters were derived from the experimentally observed updating patterns, including the observed probability of escalation and the proportions of unconditional rule followers and rule breakers. Full parameter values and implementation details are provided in the supplementary material; simulation code is available in the public repository associated with this paper [59].

### (b) Behavioural experiment

#### (i) Participants

On 1 November 2024, 4 days before the Trump–Harris American presidential election, we recruited 750 participants (from Prolific, at least 18 years of age, location: USA; *M*_age_ = 41.92, s.d. = 12.83; 49.6% male; 49.6% female; 0.8% other); 371 identified as Republicans (of whom 36% strongly) and 379 identified as Democrats (35% strongly). Eligibility required US residence, English as a first language and self-identification as either Republican or Democrat. Further recruitment and sample details are provided in the supplementary material.

#### (ii) Behavioural task

We measured rule-following using an animated task in which participants selected one of 100 horizontally arranged squares. A ‘shooting star’ animation moved from left to right, briefly lighting up squares in red (figure 2A). In each trial, participants were asked to select the square that lit up most intensely—the ‘cue’ square, which appeared between positions 50 and 70. For a stimulus video, see [59].

**Figure 4.**
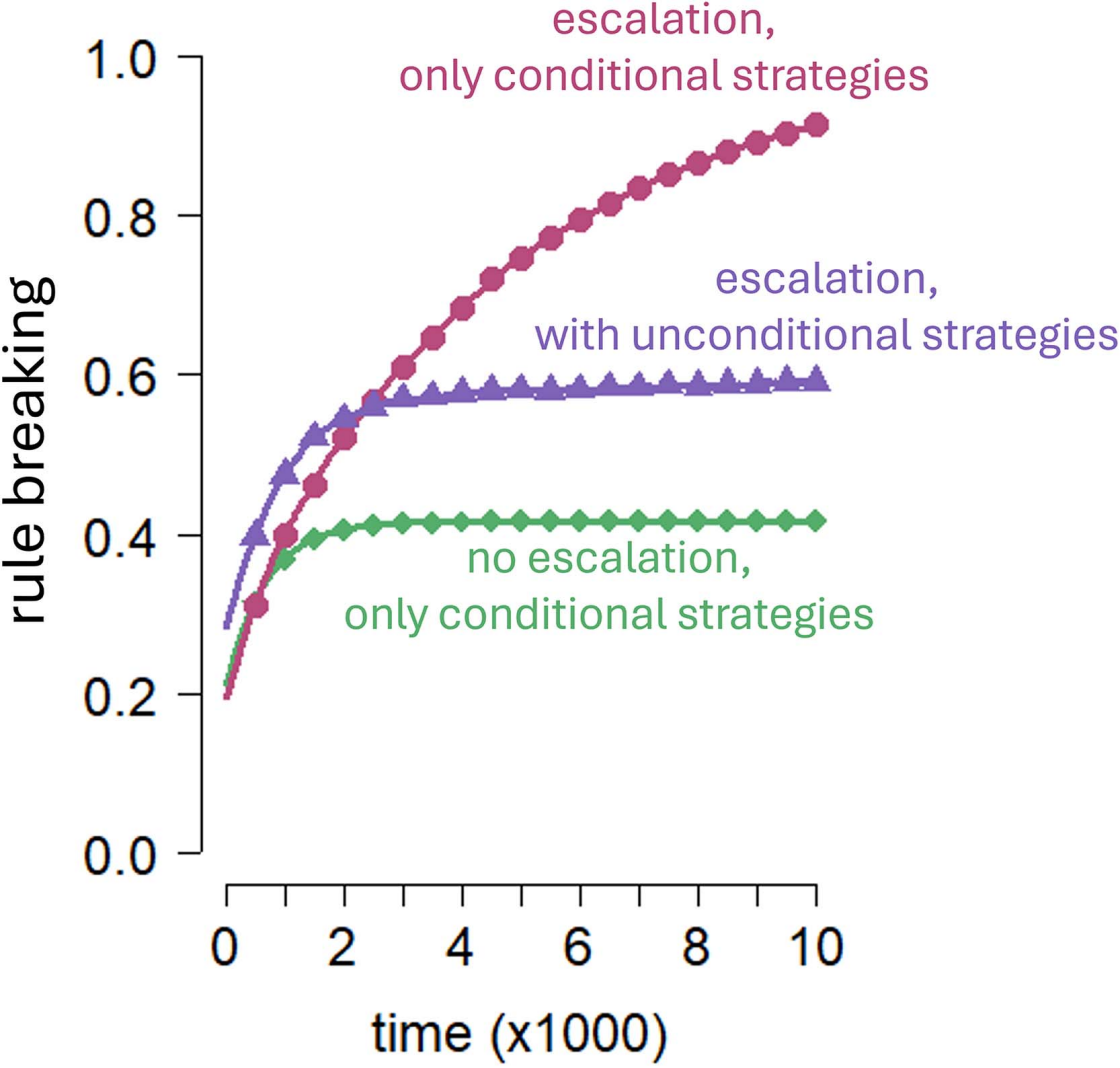
Simulated group dynamics using parameters from the experiment. Agents updated their behaviour based on the mean adjustment observed in response to rule-following and rule-violating in the experiment (green diamonds). Adding a small probability of escalation (4.4%, cf. figure 3B) amplifies rule-breaking over time (pink dots). Including a proportion of unconditional rule-followers and rule breakers, as observed in the experiment, tempers this escalation and stabilizes the group at an intermediate level of rule-breaking (blue triangles). Lines show averages; see figure S6 for individual simulation runs and figure S7 in the supplementary material for simulations using responses from the first trial as initial conditions.

After six practice trials designed to familiarize them with the stimulus (supplementary material), participants completed the six-trial main task. Participants were told that the rule was to choose the square that lit up most intensely. However, choosing farther to the right yielded higher monetary earnings, indicated by linearly increasing numbers below the squares (figure 2A). Thus, the task deliberately created a conflict between monetary incentives and an explicit rule, allowing us to study voluntary rule-following in the absence of formal enforcement. The graded response scale allowed participants to commit rule violations of varying severity. In addition, the stimulus was deliberately ambiguous, with squares adjacent to the cue square also lit, but to a lesser degree, so small deviations from the most intensely lit square were less clear-cut than larger violations. In each trial, participants first made a first choice after viewing the animation. They then saw a prerecorded choice of a prior participant (the ‘other’, presented alongside their own), and made a second choice.

#### (iii) Treatments

We employed a 2 (social proximity: ingroup vs. outgroup) × 3 (other's behaviour: follow, minor, major violation) within-subject design. Social proximity was based on political identity: participants identified as either Democrats or Republicans, and observed others were labelled accordingly. All observed choices were genuine responses from previously tested participants; no deception was used. Observed others either followed the rule (choosing a square near the cue), committed a minor rule violation (choosing a square one-third of the way between the cue and the rightmost square: cue + ⅓ * (100 − cue)) or a major rule violation (choosing square 100). Participants completed two counterbalanced blocks of three trials, one for each level of social proximity (i.e. the other's party). Within each block, they encountered the three types of other's behaviour in randomized order. See the supplementary material for procedural details, manipulation checks and screenshots of all materials.

At the end of the experiment, one choice was randomly selected for payment, where 100 points were worth 1 GBP, which was paid as a bonus on top of a flat fee of 1 GBP. Overall, the task took about 8 minutes, and on average, participants earned GBP 1.70 in total.

#### (iv) Statistical analysis

Choices were normalized as *r* = (chosen square − cue) / (100 − cue), such that *r* ≈ 0 indicates rule-following and *r* = 1 full rule-breaking. We analysed second choices using linear mixed-effects models with participant as a random intercept and first choice as a predictor. To test the preregistered asymmetry between responses to rule-following and rule-violating examples, we fixed the slope for first choice to 1, deviating from the preregistered specification to enable the planned Wald comparison. Additional analyses and model details are provided in the supplementary material. The preregistration, data and analysis code are available in the associated repository.

## Supplementary Material



## Data Availability

All data and code associated with this work are available from OSF [59]. Supplementary material is available online [70].
