# Supplementary material for: Contagion and escalation of rule violations
Source: Proc Biol Sci. 2026 Oct 21;293(2081):20261940. doi: 10.1098/rspb.2026.1940 (PMC13682926; doi:10.1098/rspb.2026.1940)
Supplement: Supplementary file 1 [file rspb.2026.1940.zip › rspb.2026.1940/rspb20261940_si_001.pdf]

# Supplementary Information

for
Contagion and escalation of rule violations

by
Lucas Molleman, Karlijn Hoyer, Gerben A. van Kleef, Christin Scholz, Jan Hausfeld

*Proceedings of the Royal Society B: Biological Sciences*
Main text DOI: 10.1098/rspb.2026.1940

**Contents**

- Supplementary Methods
- Supplementary Figures S1-S9
- Supplementary Tables S1-S4
- Screenshots of the Experimental Task
- Supplementary References

# Supplementary Methods

## Simulation details

We simulated a population of 100 agents making repeated choices on a scale from 0 (full rule compliance) to 1 (full rule breaking). Initially, choices were drawn from a normal distribution with mean 0 and standard deviation 0.5 and winsorised at 0 and 1. At each time step $t$, one focal agent was randomly selected and observed the behaviour $X$ of another randomly selected agent. The focal agent updated its choice $r$ according to $r_{t+1} = (1 - s) * r_t + s * X$, where $s$ denotes the strength of social influence. If $s = 0$, the focal agent retained its previous choice; if $s = 1$, it fully adopted the observed behaviour. We distinguished between influence from examples that were more rule-following than the focal agent ($X < r_t$; denoted $s_{follow}$) and examples that were more rule-violating ($X > r_t$, denoted $s_{violate}$).

For Fig. 1A, we contrasted a symmetric condition ($s_{follow} = s_{violate} = 0.3$) with an asymmetric condition in which rule-violating examples had greater influence ($s_{follow} = 0.2$, $s_{violate} = 0.4$). For Fig. 1B, social proximity increased influence strength ($s_{follow,\ distant} = 0.2$, $s_{follow,\ close} = 0.25$, $s_{violate,\ distant} = 0.4$, $s_{violate,\ close} = 0.5$). For Fig. 1C, inspired by [1], influence was symmetric under high social proximity but asymmetric under low social proximity ($s_{follow,\ close} = s_{violate,\ close} = 0.5$, $s_{follow,\ distant} = 0.2$ $s_{violate,\ distant} = 0.4$).

For Fig. 1D, we introduced escalation. When an agent observed a rule violation, we first drew a potential escalating choice uniformly between $X$ and 1. With probability $e$, this escalating choice was implemented. Otherwise (probability $1 - e$), the agent updated according to $s = (0.4 - s_{escalate} * e) / (1 - e)$, thereby keeping average responses to rule-violating examples at $s_{violate} = 0.4$. For Fig. 1D, we set $e = 0.5$ and $s_{follow} = 0.2$.

For the simulations in Fig. 4, we used experimentally observed parameters. In the no-escalation scenario, agents used the mean observed responsiveness to examples that were more rule-violating ($X > r_1$; $s_{violate} = 0.318$) or more rule-following ($X < r_1$; $s_{follow} = 0.126$; Fig. 4, green diamonds) than their first choice. The escalation scenario additionally used the experimentally observed escalation probability ($e = 0.044$) and the same escalation implementation as above (Fig. 4, pink dots). The final scenario also incorporated unconditional rule followers and rule breakers in the proportions observed in the experiment (Fig. 4, purple triangles). Unless otherwise specified, results were averaged across 30 simulation runs; see Figs. S1 and S6 for examples of single runs. Simulation code (written in *R*) is available at https://osf.io/xudpf.

## Experimental details

**Recruitment and participants.** Separate recruitment sessions were run for Republicans and Democrats, and the study description explicitly stated this eligibility criterion. Participants were again asked to indicate their political affiliation at the start of the study; those selecting "neither" would have been directed to a short exit screen before beginning the experiment, but in practice, no participants selected this option.

Social information came from 100 pre-recorded participants recruited from Prolific in the week prior to the main experiment. These participants identified as either Democrats or Republicans and completed the same task as participants from the main experiment, without observing social information. For each political group, we preselected responses that were as close as possible to three target levels on the normalized response scale: rule-following ($X = 0$; corresponding to the cue), minor rule violation ($X = 1/3$; one-third of the distance from

the cue to the rightmost square), and major rule violation ($X = 1$; corresponding to the rightmost square). These preselected responses were then used as the social information shown in the main experiment. Thus, all observed choices were genuine responses from prior participants and no deception was used.

As a manipulation check for social proximity, we administered the Inclusion of the Other in the Self scale [2,3], in which participants of the main experiment rated how close they felt to Democrats and Republicans on a 1 to 7 scale. Participants reported feeling closer to others of their own party (Democrats: own 4.78, other 1.67; Republicans: own 4.91; other 1.82; Fig. S2), confirming that the proximity manipulation was effective.

Before the main task, participants completed six practice trials without social information and were rewarded based on accuracy—the closer their choice to the cue, the more they earned. This familiarized them with the task and allowed us to detect baseline choice biases (e.g., due to left-to-right stimulus movement; Fig. 2A; see Fig. S8 for analysis of practice trials).

As a validation of the experimental task, we conducted a pilot study prior to the main experiment, with 100 student participants. We aimed to test whether rule-breaking in our shooting star task without social information positively correlates with lying in an established task (Fig. S9), as was previously found with a different rule-following paradigm [4].

**Data exclusions.** For the analyses, we omitted one participant who clearly did not understand their task (they consistently clicked squares below 15, far left of the cue). Including the participant in the analyses did not qualitatively change the results.

**Statistical analysis: additional details.** Our main dependent variable was participants' second choice, after observing social information. Choices were normalized as $r$ = (chosen square − cue) / (100 − cue), such that $r \approx 0$ indicates rule-following and $r = 1$ full rule breaking. Treatment effects were estimated using linear mixed-effects models with participant as a random intercept and first choice as a predictor. Models were fitted using the R package *lme4* [5].

To test the preregistered hypothesis that rule-violating examples increase rule-breaking more than rule-following examples decrease it, we deviated from the preregistered model specification by fixing the coefficient of first choice to 1. This makes the model coefficients reflect changes from participants' first to second choices and allows the effects of rule-following and rule-violating examples to be compared directly. We tested the asymmetry between these effects using a Wald test. We additionally compared the absolute magnitude of participants' updates when the observed example was more rule-following than their first choice ($X < r_1$) versus more rule-violating ($X > r_1$), again using a Wald test (reported in the main text).

To test whether social proximity moderated responses to rule-following and rule-violating examples, we added social proximity and its interactions with example type (Table 1, Models 2–3). Social proximity was coded 0 when the observed other belonged to the opposing political party and 1 when they belonged to the participant's own party. We repeated Model 3 using social proximity as a continuous predictor, based on participants' Inclusion of the Other in the Self (IOS) ratings of members of the observed other's political party (Table S2).

As a robustness analysis, we repeated the treatment analysis using only participants' first experimental trial, thereby eliminating any potential carry-over effects from exposure to examples in preceding trials (Table S3).

**Consistency in rule-related behaviour.** To assess participants' consistency in rule-following and rule-breaking, we used two alternative specifications. First, participants were classified as consistent rule followers (breakers) if all their responses satisfied $r_1$, $r_2$ <0.1 (0.9) across all trials. Second, for each participant we calculated the maximum deviation observed during the accuracy block and determined the proportion of responses $r_1$ and $r_2$ exceeding this individual threshold to the right (i.e. self-serving rule violations). The two approaches capture somewhat different notions of consistency and therefore produce the ranges reported in the main text.

## Supplementary Figures

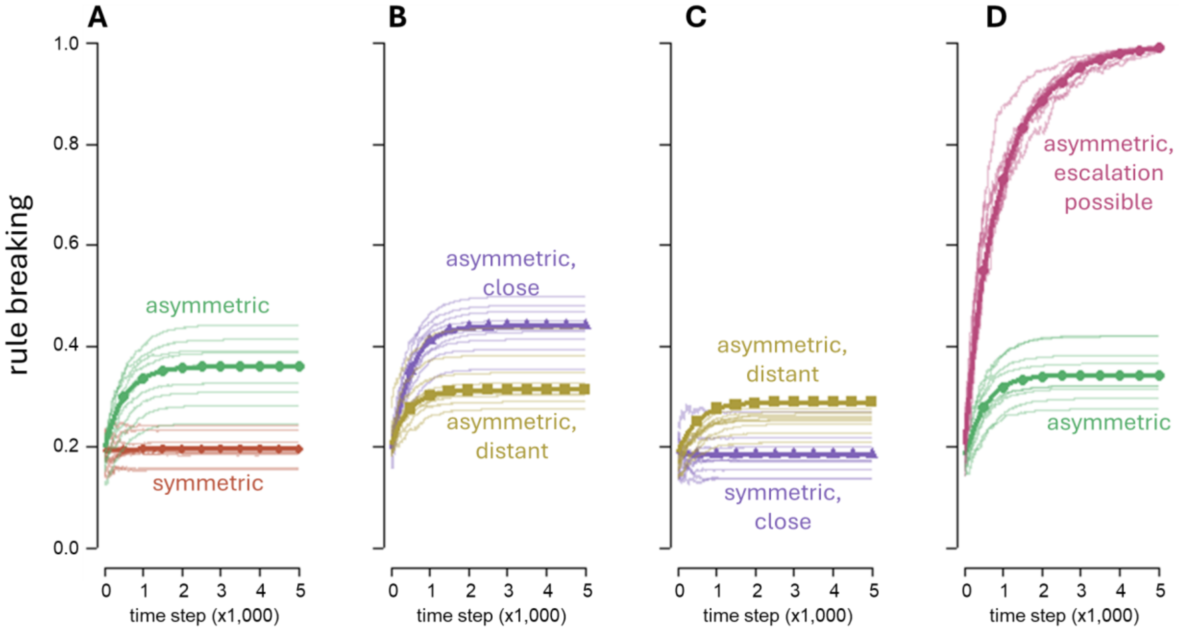


**Fig. S1.** Simulations from Fig. 1, with the additional inclusion of 10 individual runs sampled from the 30 runs per scenario.

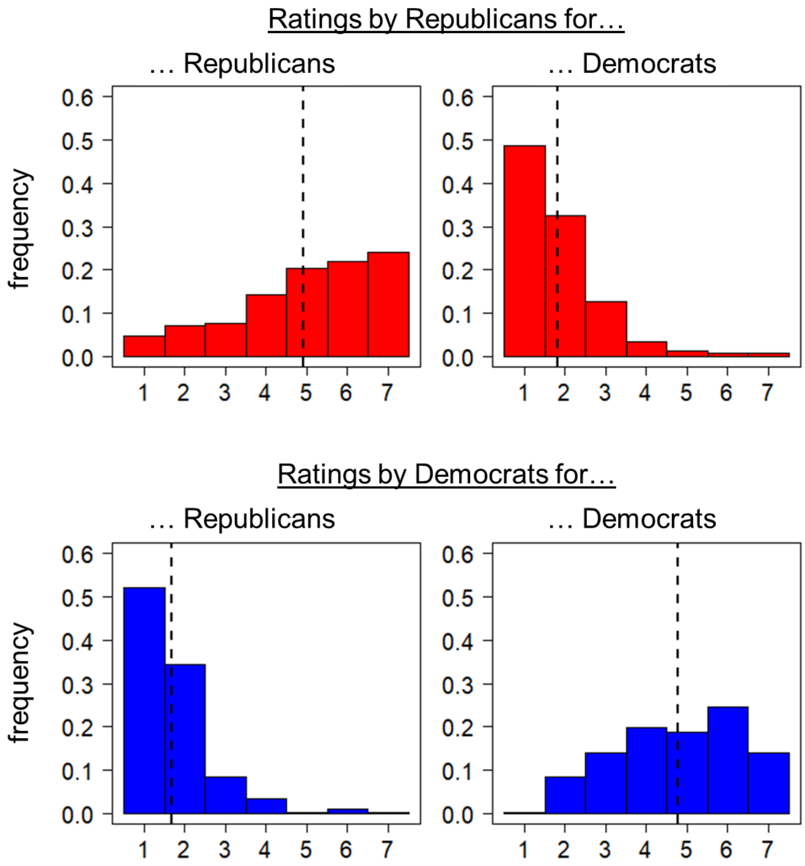


**Fig. S2. Manipulation test for social proximity.** Participants filled out the Inclusion of Others in the Self Scale [2,3], answering on a 1-7 scale to the question “Which picture best describes your relationship with Republicans / Democrats?”, where 1 indicated the lowest social proximity, and 7 the highest. Both Republicans and Democrats rated members of their own political party much higher than that of the other party (paired t-tests for ratings by Republicans: $t(370)=30.23$, $p<0.001$; for Democrats: $t(376)=34.19$, $p<0.001$), indicating that our manipulation for social proximity was successful. Vertical dashed lines in the panels show means.

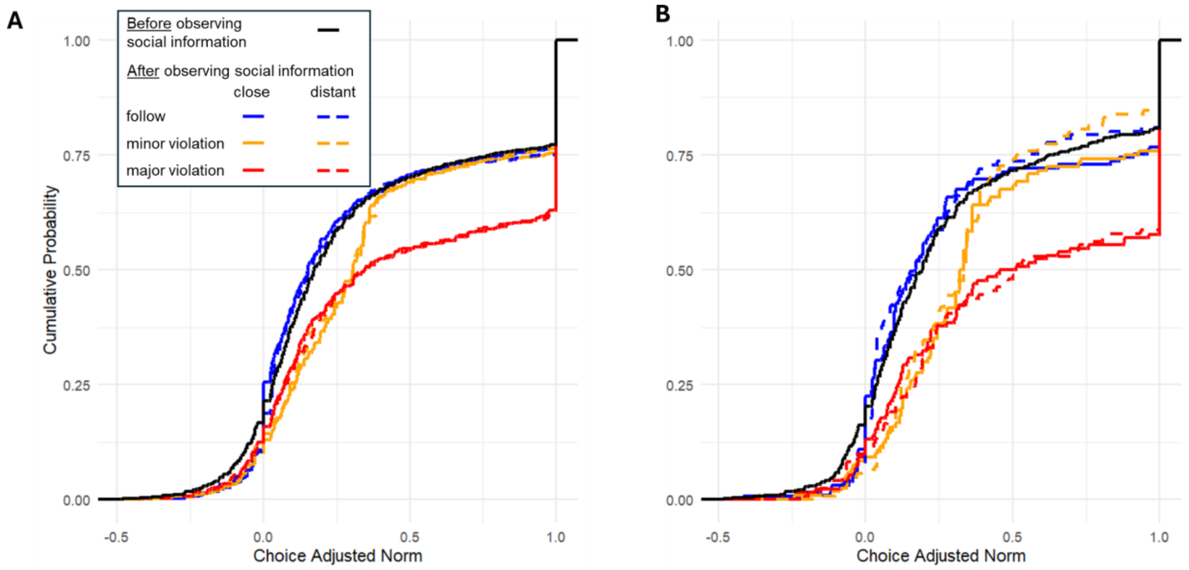


**Fig. S3.** Cumulative distributions of choices across treatments. Black lines indicate first choices, before observing social information. Coloured lines show second choices broken down by treatment. We observe that the type of example (rule-following, minor violation, major violation) has strong effects, and social proximity has virtually no effect (indicated by the solid and dotted lines of the same colour being virtually indistinguishable). Panel **A** shows data from all trials (cf. Fig. 2B), panel **B** shows choices trial 1 only (so no spill-over effects could play a role). For a regression fitted to responses to social information in trial 1 only, see Table S3.

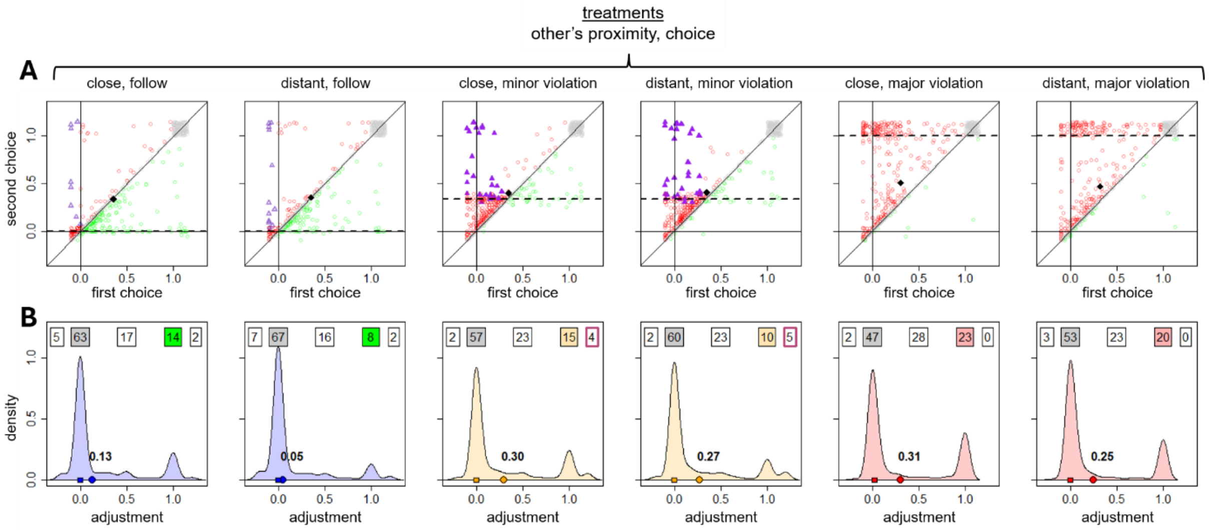


**Fig. S4.** Choices and adjustments split out by social proximity and the example's choice. Panels are the same as in Fig. 3 of the main text, but now broken down by low vs high social proximity. **A.** Scatterplots show second choices as a function of first choices when the observed other participant either followed the rule, or committed minor or major violations. Dots show participants' choices in individual trials. Colours indicate the types of updates (green: reduced rule-breaking; red: increased rule-breaking; grey: no change; purple: updates exceeding the observed behaviour ($r_1 < X$ and $r_2 > X$). In the rule-following conditions, open triangles denote participants whose first choice lay left of the cue and whose second choice overshot the observed rule-following example; in the minor violation conditions, filled triangles denote "escalation" beyond the observed rule violation. Thick horizontal dashed lines show average social information ($X$) in each treatment. Thin lines at x=0 and y=0 indicate the position of the cue (full rule-following in first and second choices). The diagonal indicates no change. First or second choices that are full rule breaking (x=1 and y=1) are shown with positive-only jitter to reduce overplotting. **B.** Distributions of adjustments from first to second choices calculated as $s=(r_2 - r_1) / (X - r_1)$. Circles with labels indicate average adjustments, and squares indicate medians. For visual presentation we winsorised the data at -1.2 and 1.2. Percentages of qualitatively different adjustment types are shown up top, with from left to right: moving away from social information ($s$<0), staying ($s$=0), compromising (0<$s$<1), copying ($s$=1) and escalating ($s$>1).

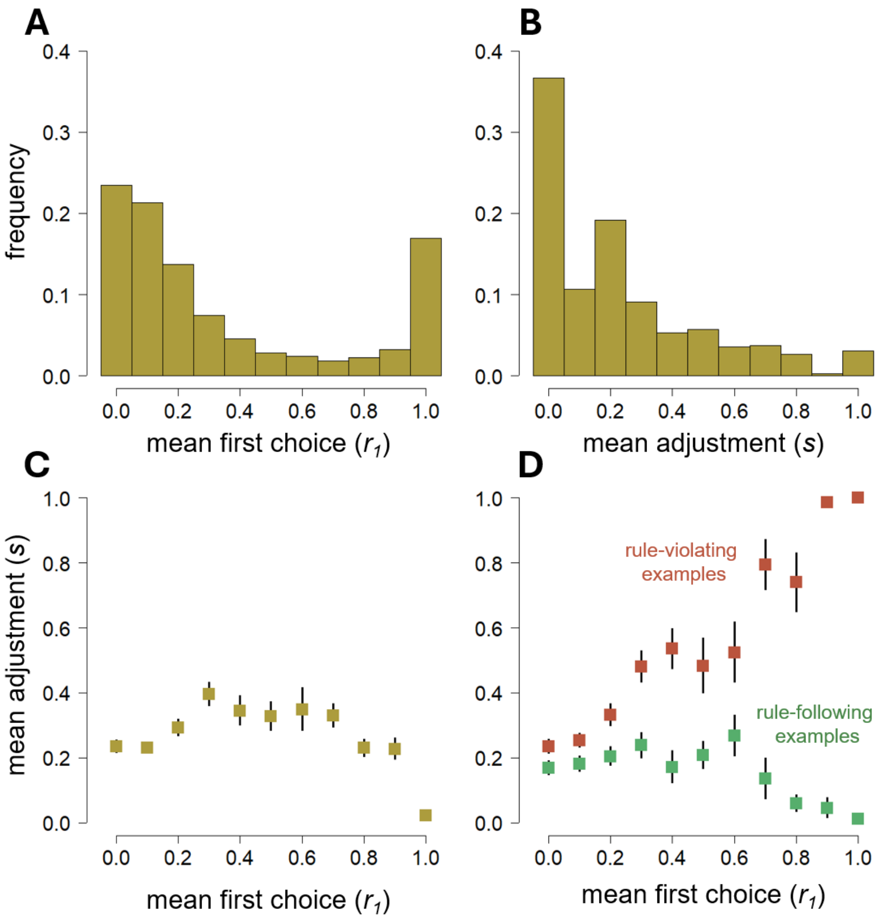


**Fig S5.** Individual-level summary statistics, averaged across the six trials of the task. **A.** Mean first choices. Before analysis, values were left-winsorised at $r$=0. **B.** Distribution of adjustments ($s$), where $s$=0 indicates no update (so that $r_2$=$r_1$), and $s$=1 indicates full copying of the social information; see Fig. 3B and Fig. S4B for breakdowns by treatment. **C.** Mean adjustment (s) as a function of mean first choices. The hump-shaped pattern indicates that adjustments tended to be largest for individuals who tended to have intermediate first choices. **D.** Mean adjustments (s) as in C, now broken down by the social information being more rule-following ($X$<$r_1$) or more rule-violating ($X$>$r_1$) than the participant's first choice. Note that at the extremes (e.g., at x=1), rule-violating examples tended to be very close to the participants' first choice, so already minor adjustments (in absolute terms) led to high values of $s$ (red squares on the right hand side).

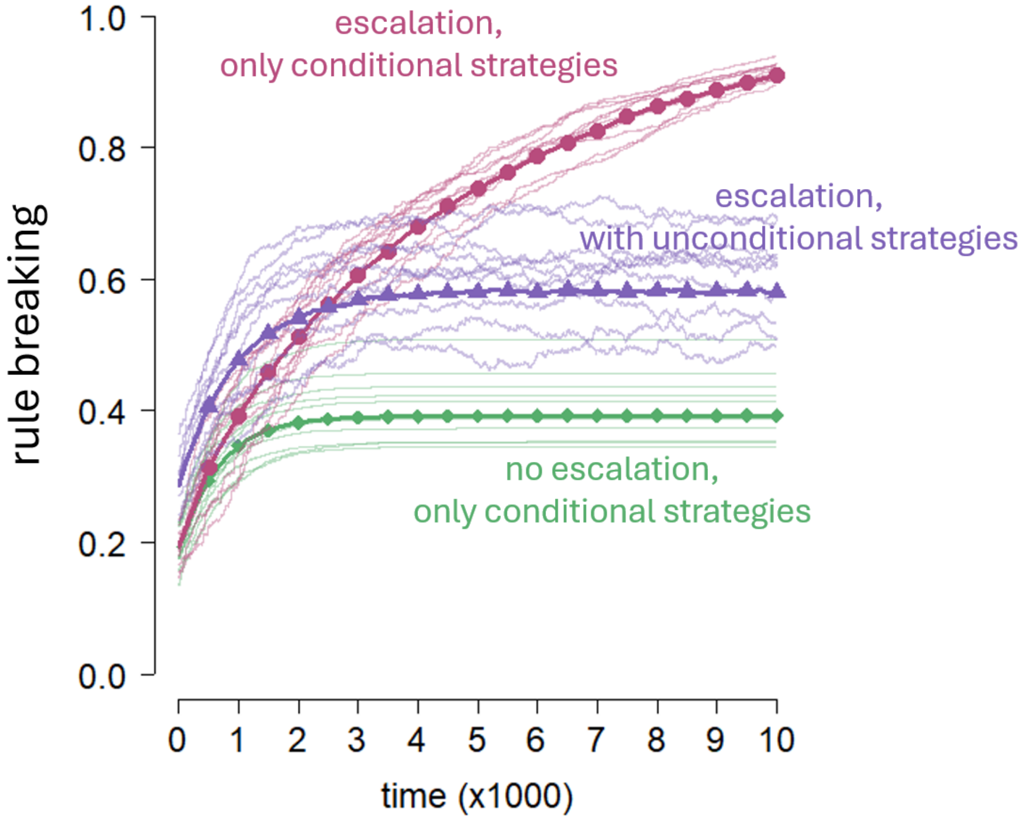


**Fig. S6. Simulations parameterised with experimentally-observed values.** The simulations are identical to those presented in Fig. 4 of the main text, but now include 10 individual runs sampled from the 30 runs per scenario. For simulations using first choices from the first experimental trial as initial conditions, see Fig. S7. For simulation code, see the publicly accessible repository associated with this paper: https://osf.io/xudpf.

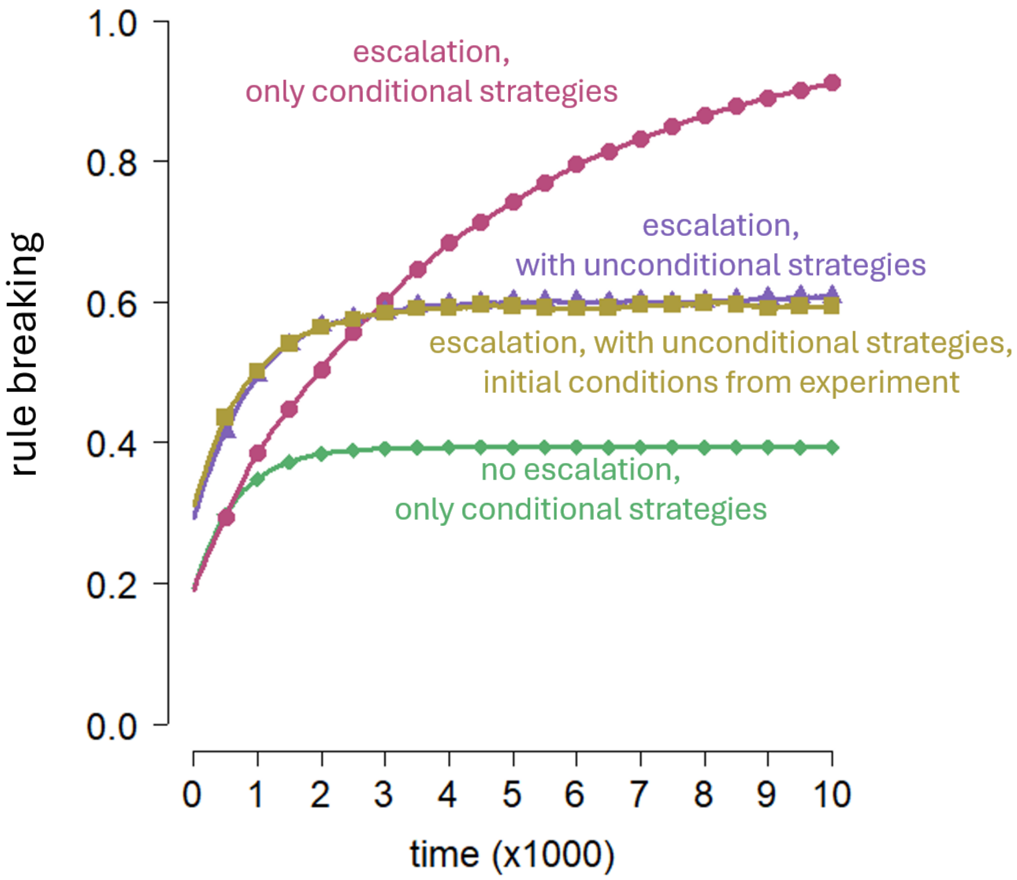


**Fig. S7. Simulations parameterised with experimentally-observed values, including the initial conditions sampled from the first trial.** For each simulation run, we sampled 100 experimental participants and used their first choices in the first trial of the rule-following task to initialize the simulation. For illustration purposes and to avoid cluttering, we omit the lines of individual simulation runs (cf. Fig. S6), and show average levels of rule breaking from 30 simulation runs instead. For simulation code, see the repository associated with this paper: https://osf.io/xudpf.

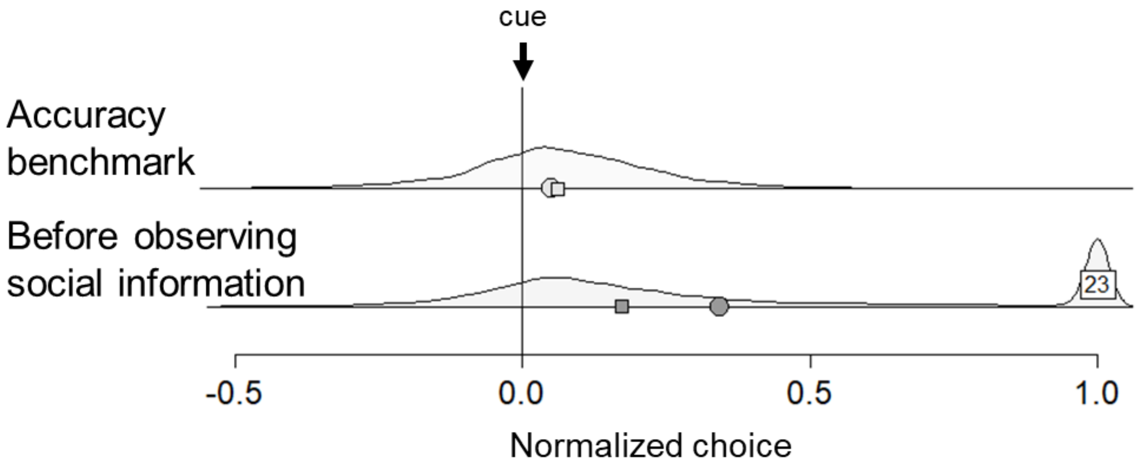


**Fig. S8. Responses in the accuracy block and in the "rule" block before observing social information.** When incentivised for accuracy participants overwhelmingly chose squares near the cue square, with a slight but significant bias to the right, i.e. the direction of the shooting star (Fig. 2, top grey dot: $r_1$=0.051; test against 0: random effects model with "participant" as random intercept: t=7.39, p<0.001). First choices in the rule block were further to the right than in the accuracy block (mean $r_1$=0.334; b=0.283, t=46.744, p<0.001; Fig. S2). If we omit the trials in which participants violated the rule outright in their first choice ($r_1$=1; the hump at x=1 in the ridgeplot), we still observe that the distribution was shifted to the right in the rule block relative to the accuracy block (b=0.078, t=19.287, p<0.001).

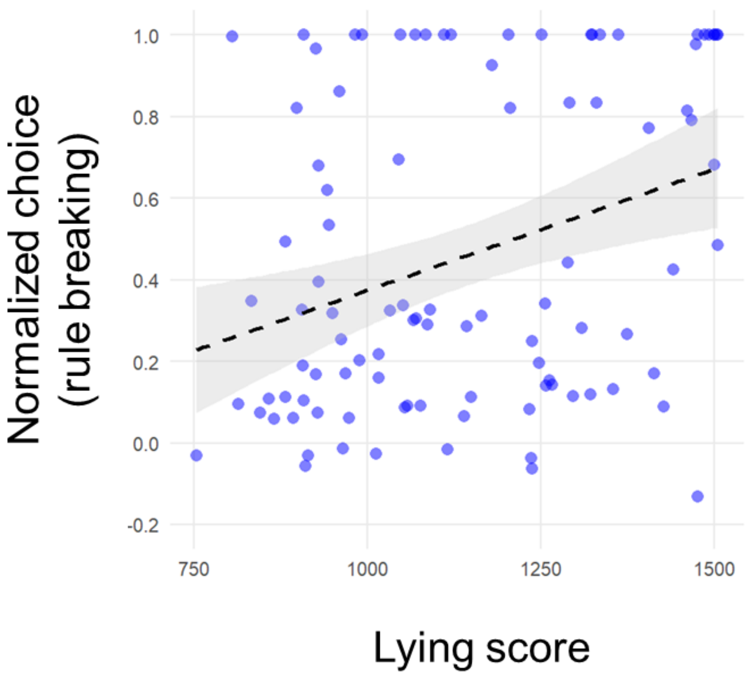


**Fig. S9.** Rule breaking in the star task is predicted by lying behaviour. Plots are based on data from a student sample from Czechia who completed the experiment in a physical laboratory (N=98; 31 male, 66 female, 1 other; mean age: 23.6, s.d, 6.1). Participants completed five trials of the star task (Methods) without social information. Their average normalized response is shown on the y-axis. Prior to the star task, they completed a lying task similar to the die-under-cup paradigm [6]. In each of 24 trials, participants saw 6 letters and were told to memorize one of them. Then, the letters were matched to points ranging between 10 and 95 (an example of a trial would be C=10, B=15, E=20, A= 25, F=30, D=35). Participants had to indicate the letter they memorized, and earned the points associated with the chosen letter. Importantly, participants could lie about which letter they memorized, and the experimenter could not find this out. The score on the x-axis in the figure refers to the total number of points of participants across the 24 trials. The expected score of an honest person was up to around 1200 points. The maximum possible score was 1504, with higher scores likely reflecting increasing dishonesty. Overall, degrees of rule breaking were higher in this lab sample than in the online sample of U.S. Prolific workers (mean normalized choice indicating the degree of rule breaking: 0.466 in this sample, versus 0.342 in the American sample; in a linear mixed effects model with “participant” as random intercept, the fixed effect of “country” as the only predictor was 0.155, with t=-9.776, p<0.001). Crucially, we observe a positive correlation between the lying score (x-axis) and the mean degree of rule breaking across the five trials (ρ=0.327, t = 3.39, p = 0.001), which is consistent with previous findings [4]. This correlation was robust to only including the first period of the star task (ρ=0.253, t = 2.564, p = 0.012). These results suggest that our rule-following task captures psychological mechanisms that also drive behaviour in related domains.

## Supplementary Tables

| Predictor | Estimate | Estimate | Estimate | Estimate |
|---|---|---|---|---|
| Intercept (gender=male, party=Republican) | b=-0.003 (-0.051 0.046), t=-0.103, p=0.918 | b=0.060 (0.041 0.079), t=6.124, p<0.001 | b=-0.017 (-0.063 0.029), t=-0.718, p=0.473 | b=0.057 (0.038 0.076), t=5.848, p<0.001 |
| Gender=female | b=-0.022 (-0.049 0.005), t=-1.565, p=0.118 | b=-0.016 (- 0.043 0.011), t=-1.179, p=0.239 | | |
| Gender=other | -0.103 (-0.254 0.049), t=-1.329, p=0.184 | -0.115 (-0.267 0.037),t=- 1.488,p=0.137 | | |
| Age | 0.002 (0.001 0.003), t=3.116, p=0.002 | | 0.002 (0.001 0.003), t=3.027, p=0.003 | |
| Democrat | -0.010 (-0.037 0.017), t=-0.750, p=0.454 | | | -0.013 (-0.040 0.014), t=-0.916, p=0.360 |

**Table S1. Determinants of rule-breaking prior to observing social information.** The dependent variable in the regressions was participants' normalized first choices. All estimates are from mixed linear models with "first choice" as dependent variable, and with "participant" as randomly varying intercept.

| Predictor | Estimate |
|---|---|
| Following example (baseline) | b=-0.004 (0.012), t=-0.356, p=0.722 |
| Major violation example | b=0.131 (0.015), t=8.483, p<0.001 |
| Minor violation example | b=0.056 (0.015), t=3.612, p<0.001 |
| Social proximity (measured as IOS score) | b=-0.002 (0.003), t=-0.802, p=0.422 |
| Major violation example x IOS | b=0.017 (0.004), t=4.161, p< 0.001 |
| Minor violation example x IOS | b=0.003 (0.004), t=0.647, p=0.517 |

**Table S2. Model 3 from Table 1 (main text) with social proximity as a continuous predictor based on the Inclusion of the Other in the Self (IOS) scale** [2,3]**.** Shown are estimates (with standard errors in brackets) from a linear mixed model fitted to second choices (after observing social information), with "participant" as randomly varying intercept. As in the model presented in the main text, we observe that examples committing major violations particularly increase rule-breaking when the example is set by a socially close other (interaction effect "Major violation example x IOS").

| Predictor | Estimates |
| --- | --- |
| Following example (baseline) | b=-0.002 (0.027), t=-0.089, p=0.929 |
| Major violation example | b=0.224 (0.038), t=5.915, p<0.001 |
| Minor violation example | b=0.057 (0.038), t=1.499, p=0.134 |
| Social proximity (sp) | b=-0.028 (0.037), t=-0.763, p=0.446 |
| Major violation example x sp | b=0.082 (0.052), t=1.586, p=0.113 |
| Minor violation example x sp | b=0.038 (0.052), t=0.736, p=0.462 |

**Table S3. Model 3 from Table 1, based on the first trial only.** Shown are estimates (with standard errors in brackets) from linear regression fitted to second choices (after observing social information). As for the regressions in the main text, we fixed the slope of the participant's first choice (before observing social information) to 1.

| Predictor | Estimates |
| --- | --- |
| Following example (baseline) | b=-0.024 (0.028), t=-0.848, p=0.396 |
| Major violation example | b=0.134 (0.035), t=3.853, p<0.001 |
| Minor violation example | b=0.047 (0.035), t=1.343, p=0.179 |
| Social proximity (sp) | b=-0.028 (0.035), t=-0.779, p=0.436 |
| Major violation example x sp | b=0.108 (0.049), t=2.193, p=0.028 |
| Minor violation example x sp | b=0.038 (0.052), t=0.736, p=0.462 |
| Partisan strength (coded as 0=low, 1=high) | b=0.016 (0.019), t=0.837, p=0.403 |
| Partisan strength x sp | b=0.004 (0.024), t=0.173, p=0.862 |
| Partisan strength x Major violation example | b=0.012 (0.024), t=0.477, p=0.634 |
| Partisan strength x Minor violation example | b=0.007 (0.024), t=0.290, p=0.772 |
| Partisan strength x sp x Major violation example | b=-0.025 (0.034), t=-0.740, p=0.459 |
| Partisan strength x sp x Minor violation example | b=-0.022 (0.034), t=-0.646, p=0.519 |

**Table S4. Model 3 from Table 1, including an interaction term with "partisan strength".** This added predictor measured participants' self-rating on whether they saw themselves as a strong (coded as 1) or not a strong (coded as 0) Republican or Democrat. We do not observe any moderating effects of this predictor.

## Screenshots of Experimental Task

Below we show the experimental materials as shown to participants.

Where appropriate, we add comments in grey boxes like this one.

### Welcome

In this study, you will play **two short games**. Before and afterwards, we will ask you a few questions.

Completing the study will take about 8 minutes.

You will receive a participation fee of **£1.20**, and can earn a **bonus** payment of **up to £1.00** depending on your decisions in the game.

Continue

### Start questionnaire

Please answer the questions below.

What is your gender?

male | female | other / don't want to say

What is your age?

Generally speaking, I think of myself as a

Republican | Democrat | Neither

If participants chose “Neither” and confirmed that choice, they would have been directed to a screen ending the study (but in practice, no participant chose that option).

After filling in the political affiliation “Republican” or “Democrat”, another question appeared on the same screen:

Generally speaking, I think of myself as a

Republican | Democrat | Neither

Would you call yourself a strong Republican or a not very strong Republican?

Strong | Not very strong

Continue to task

## What you need to know:

You will play **two short games**.
Each game lasts 6 rounds.

In each game you can earn points.

At the end of the study, **the computer will select 1 of your decisions in this study** to determine your bonus payment.
Any bonus you earn comes on top of your participation fee of £1.20.

100 points are worth £1.00.

Continue

## Instructions game 1:

In each round, you will see a line of squares:

Click the "Show flash!" button and closely watch what happens. A flash will briefly light up some of the squares in red.

Your task is to *click on the square that lit up most intensely*.

**Your earnings in game 1**

If you choose the square that lit up *most intensely*, you will earn 100 points.
For each square you are off, 10 points are subtracted. Your points in a round cannot become negative.

You can now test the "Show flash!" button so you know what to expect. Now, you can press it as often as you like, but in the game itself you will see each flash once. So watch closely!

Show flash!

Continue

Clicking the purple button activated the animation, which moved from left to right on the instruction screen. In this instruction screen, participants could click it as many times as they wanted (they had to click it at least once to proceed). In the tasks themselves, they could see the animations only once.

Below you find a screenshot in the middle of the animation. For a movie, see https://osf.io/xudpf.

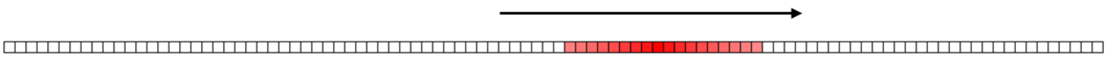

Control questions followed:

Please answer the following questions to check if you understand your task.

*Remember:* The maximum number of points in a round is 100. For each square you are off, 10 points are subtracted. Your points in a round cannot become negative.

The square that lit up most intensely was square 60. You selected square 62. How much do you earn?

The square that lit up most intensely was square 77. You selected square 90. How much do you earn?

Back to instructions

Continue

**That's correct! Let's start the first game.**

Continue

Then participants completed 6 rounds of the task in which they were rewarded for accuracy.

Show flash!

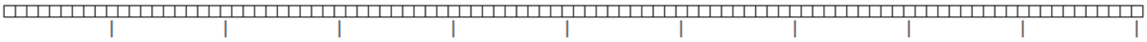

Select a square by clicking on the line of squares.

**Which square lit up most intensely?**

Continue

For illustration purposes, we magnified the mouse cursor.

Show flash!

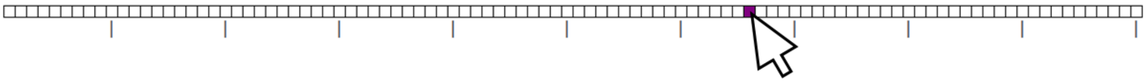

Select a square by clicking on the line of squares.

**Which square lit up most intensely?**

Continue

Round 1 of 6 completed.

Continue

## Instructions game 2:

Game 2 is similar to game 1. Again, the squares on the line will briefly light up.

In this game, your earnings from a round depend on which square you choose.

**The farther right you click, the more points you earn.**

The number of points is indicated below the row of squares:

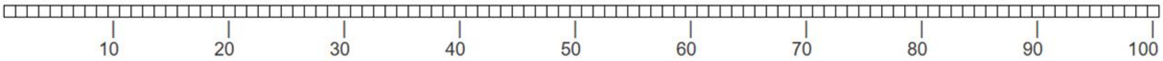


**The rule is to click the square that lit up most intensely.**

Remember: you will only see the flash once.

After you made your choice, **you will see the choice of a previous participant** on the next screen.

You then have the option to adjust your first choice.

The previous participant is either a Republican or a Democrat

Continue

Please answer the following questions to check if you understand your task.
Remember: The maximum number of points in a round is 100. The farther right you click, the more points you earn.

The square that lit up most intensely was square 60. You selected square 62. How much do you earn?

The square that lit up most intensely was square 77. You selected square 90. How much do you earn?

The rule is to click the square that lit up most intensely.

True False

Back to instructions

Continue

**That's correct! Let's start the second game.**

**Remember: the rule is to click the square that lit up most intensely.**

Continue

The first block of trials starts. Participants are informed about the political affiliation of the prior participants (“demonstrators”) they are going to observe.

In this game, we will show you which square was chosen by previous participants.

In the following three rounds, you will see the choices of three different participants identifying as

**Democrats**

Continue

This is the main task screen (for a video, see https://osf.io/xudpf). We break it down into four to illustrate the four key steps. First, participants have to press the purple button.

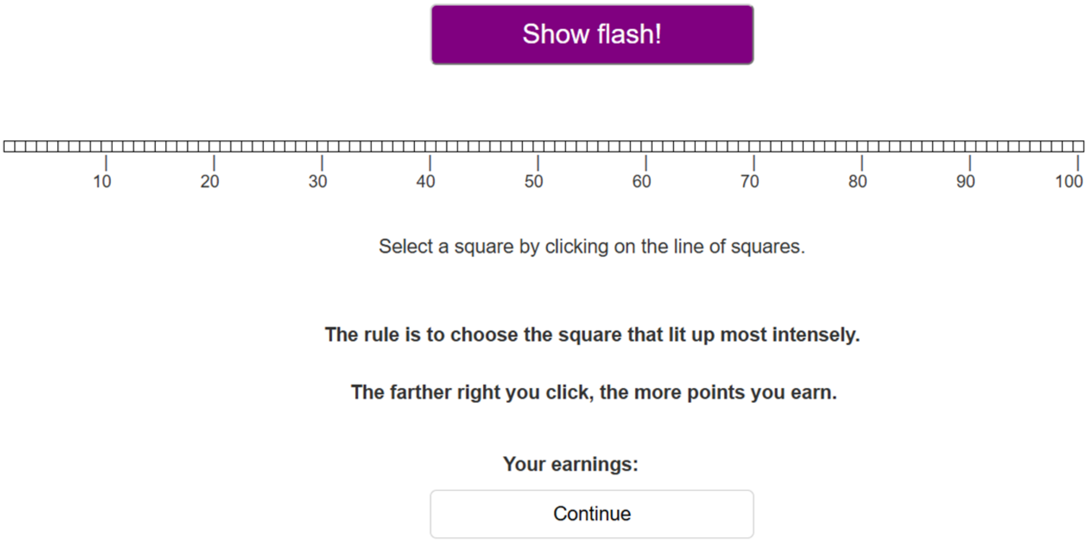


Then the animation plays, and participants can make their first choice for that trial. Again, we magnified the mouse cursor for illustration purposes. The earnings are updated upon mouse click.

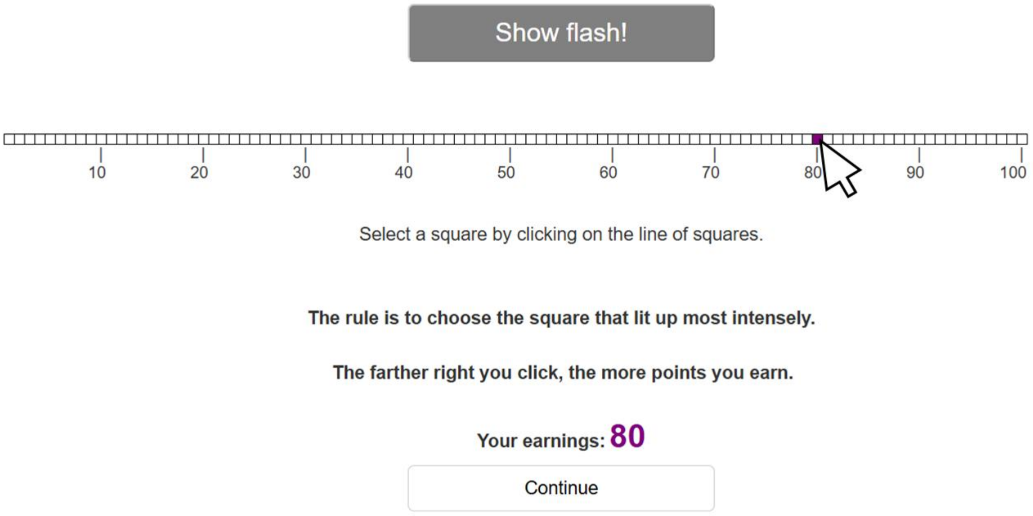


Then participants observe the decision of a prior participant, in this case a Democrat-identifying person who clicked square 75. Note that their own first choice in the current trial is shown with the purple-bordered square. Also note that in the three trials within this block, the demonstrators are indicated as Democrat 1, Democrat 2, and Democrat 3 (or Republican 1, Republican 2, and Republican 3) to reiterate that the shown choices in the three trials were made by three different demonstrators.
This screen showed the treatment variations with the demonstrator following the rule, or showing minor or major violations.

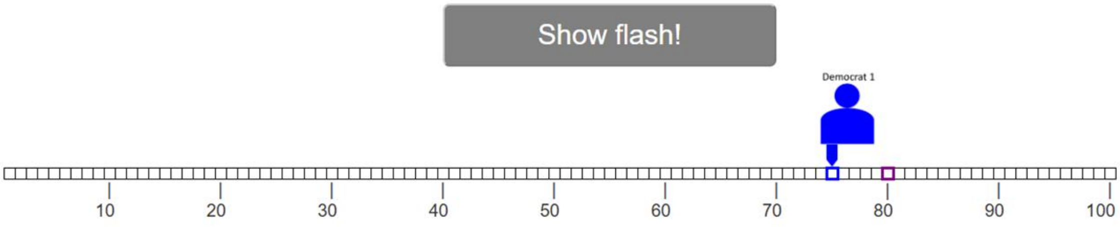


Your first click is shown in purple.

The click of the other participant is shown in blue. **You see the click of a Democrat.**

If you want, you can adjust your first click.

Select a square by clicking on the line of squares.

**The rule is to choose the square that lit up most intensely.**

**The farther right you click, the more points you earn.**

**Your earnings:**

Continue

Finally, participants make their second choice in a trial.

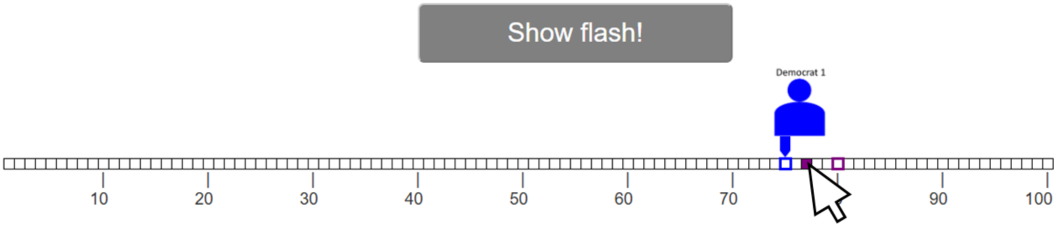


Your first click is shown in purple.

The click of the other participant is shown in blue. **You see the click of a Democrat.**

If you want, you can adjust your first click.

Select a square by clicking on the line of squares.

**The rule is to choose the square that lit up most intensely.**

**The farther right you click, the more points you earn.**

**Your earnings: 77**

Continue

Round 1 of 6 completed.

Continue

Participants complete two blocks of three trials each. After the first block has ended, participants see a screen announcing that the “demonstrators” will now be of the other political affiliation.

In this game, we will show you which square was chosen by previous participants.

In the following three rounds, you will see the choices of three different participants identifying as

**Republicans**

Continue

Then the second block of 3 trials followed, with screens looking the same as above (with the color and label of the other being accordingly changed to the political affiliation).

**Game 2 completed. Congratulations!**

To questionnaire

This is the Inclusion of the Other in the Self measure adapted from [2,3].

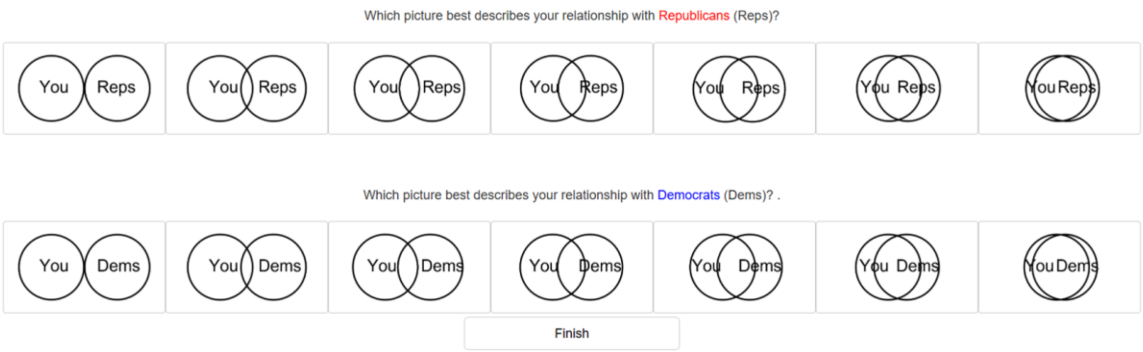


## Earnings

Your participation fee: **£1.20**

**Your bonus:**

The computer selected game 1, round 1.

In this game, more accurate choices earned you more points.
In the selected round, you were 11 square(s) off the right answer. You earned **0 points.**

As you know, in this study, 100 points are worth £1.00.
**Your total bonus is £0.00 and, hence, your total payment is £1.20**

To receive your earnings, fill out this code in Prolific

You can then close this window. We thank you for your participation.

## Supplementary References


1. Bicchieri C, Dimant E, Gächter S, Nosenzo D. 2022 Social proximity and the erosion of norm compliance. *Games Econ. Behav.* **132**, 59–72. (doi:10.1016/j.geb.2021.11.012)

2. Gächter S, Starmer C, Tufano F. 2015 Measuring the Closeness of Relationships: A Comprehensive Evaluation of the 'Inclusion of the Other in the Self' Scale. *PLoS ONE* **10**, e0129478. (doi:10.1371/journal.pone.0129478)

3. Aron A, Aron EN, Smollan D. 1992 Inclusion of Other in the Self Scale and the structure of interpersonal closeness. *J. Pers. Soc. Psychol.* **63**, 596–612. (doi:10.1037/0022-3514.63.4.596)

4. Gross J, De Dreu CKW. 2021 Rule Following Mitigates Collaborative Cheating and Facilitates the Spreading of Honesty Within Groups. *Pers. Soc. Psychol. Bull.* **47**, 395–409. (doi:10.1177/0146167220927195)

5. Bates D, Maechler M, Bolker B. 2012 lme4: Linear mixed-effects models using S4 classes.

6. Fischbacher U, Föllmi-Heusi F. 2013 Lies in disguise—an experimental study on cheating. *J. Eur. Econ. Assoc.* **11**, 525–547.
